# Association Mapping Analysis for Fruit Quality Traits in *Prunus persica* Using SNP Markers

**DOI:** 10.3389/fpls.2018.02005

**Published:** 2019-01-17

**Authors:** Carolina Font i Forcada, Verónica Guajardo, Sebastian Reyes Chin-Wo, María Ángeles Moreno

**Affiliations:** ^1^Department of Pomology, Estación Experimental de Aula Dei-CSIC, Zaragoza, Spain; ^2^Centro de Estudios Avanzados en Fruticultura, Rengo, Chile; ^3^Genome Center, University of California, Davis, Davis, CA, United States

**Keywords:** peach, germplasm, firmness, antioxidants, sugar content, single nucleotide polymorphism, candidate genes

## Abstract

The identification of genes involved in variation of peach fruit quality would assist breeders to create new cultivars with improved fruit quality. Peach is a genetic and genomic model within the Rosaceae. A large quantity of useful data suitable for fine mapping using Single Nucleotide Polymorphisms (SNPs) from the peach genome sequence was used in this study. A set of 94 individuals from a peach germplasm collection was phenotyped and genotyped, including local Spanish and modern cultivars maintained at the Experimental Station of Aula Dei, Spain. Phenotypic evaluation based on agronomical, pomological and fruit quality traits was performed at least 3 years. A set of 4,558 out of a total of 8,144 SNPs markers developed by the Illumina Infinium BeadArray (v1.0) technology platform, covering the peach genome, were analyzed for population structure analysis and genome-wide association studies (GWAS). Population structure analysis identified two subpopulations, with admixture within them. While one subpopulation contains only modern cultivars, the other one is formed by local Spanish and several modern cultivars from international breeding programs. To test the marker trait associations between markers and phenotypic traits, four models comprising both general linear model (GLM) and mixed linear model (MLM) were selected. The MLM approach using co-ancestry values from population structure and kinship estimates (K model) identified a maximum of 347 significant associations between markers and traits. The associations found appeared to map within the interval where many candidate genes involved in different pathways are predicted in the peach genome. These results represent a promising situation for GWAS in the identification of SNP variants associated to fruit quality traits, potentially applicable in peach breeding programs.

## Introduction

Peach [*Prunus persica* (L.) Batsch] is a model plant inside the family Rosaceae mainly due to its relatively small genome (∼230 Mb; Verde et al., 2017) and a low diploidy level (2n = 2x = 16), as well as its self-compatible mating system (Arús et al., 2012) and a relatively short juvenile period (2–4 years). In addition, it is one of the best genetically characterized *Prunus* species, with well-known genes controlling important traits that display Mendelian inheritance patterns, such as fruit flesh color (Y), stone adhesion-flesh texture (F-M), fruit shape (S) or sub-acidity (D; Dirlewanger and Arús, 2004). These traits have been studied in commercial and traditional peach cultivars (Cevallos-Casals et al., 2006; Reig et al., 2013, 2015; Font i Forcada et al., 2014) as well as in peach breeding progenies (Cantín et al., 2010a,b). From the market standpoint, world peach production exceeds more than twenty million tons of fruit per year, being one of the most important fruit grown in the world. The main producer countries in the world are China, Italy, Spain, and United States^1^.

In the last decades, peach diversity has been drastically reduced by the use of modern cultivars that share a few common ancestors (Aranzana et al., 2010). Thus, peach has a narrower genetic base (Scorza et al., 1985) in comparison with other species such as grape (Barnaud et al., 2006) or maize (Remington et al., 2001), where several studies (Yan et al., 2009) suggest that single nucleotide polymorphisms (SNPs) estimate a much lower decay of linkage disequilibrium (LD) than microsatellite (SSRs). However, the greater frequency of SNPs over SSRs makes the former more useful when the polymorphism within specific genes is desired for targeted investigations. The number and marker type used for investigating population structure has a significant effect on the rate of significant associations (Matthies et al., 2012; Cappa et al., 2013).

As an alternative to analysis in controlled crosses, association mapping (AM) is the non-random association of alleles at distinct loci in a sample population. AM is now being largely applied, using SSRs, to many crops, such as maize (Krill et al., 2010), potato (Pajerowska-Mukhtar et al., 2009), wheat (Breseghello and Sorrells, 2006), but only few studies have been carried out in fruit tree crops, such as peach (Font i Forcada et al., 2013), apple (Cevik et al., 2010), or pear (Oraguzie et al., 2010). This approach relies on the strength of association between genetic markers and phenotype (Mackay and Powell, 2007) and delimited genomic regions, which is helpful in locating and selecting candidate genes controlling the studied trait. In crop plants, the potential of exploiting LD in population-based association mapping, with the objective of estimating the position of a gene conferring a specific trait or phenotype by using LD between alleles of genetically mapped markers, has become a focus of considerable interest. As spurious associations between phenotypes and marker loci may be caused by population structure (Mariette et al., 2010; Ganopoulos et al., 2011), the structure and extent of LD within a sample population must be known before selecting an appropriate association mapping strategy (Lander and Schork, 1994). The number of markers available, their format, and cost currently limits whole-genome association studies in crop plants. In peach, different studies have been carried out using SSRs markers in cultivars with different genetic origin indicating that LD in this crop is quite high (Aranzana et al., 2010; Cao et al., 2012; Font i Forcada et al., 2013). In recent years, it has become increasingly common to use SNPs markers to get saturated genetic maps. SNPs have started to be used to study the whole-genome scans for diversity analysis, germplasm management, genetic fingerprinting, parentage verification, candidate genes and gene mapping in the Rosaceae family (Wu et al., 2008; Verde et al., 2012; Guajardo et al., 2015; Fresnedo-Ramírez et al., 2016; Zeballos et al., 2016; Hernández Mora et al., 2017). Multiplex SNP genotyping enables cost effective marker-assisted selection strategies, whole genome fingerprinting and genome-wide association studies (GWAS). Molecular markers are now widely employed in plant breeding for the acceleration of plant selection gains through marker-assisted selection (MAS) on the basis of individual genes or at the whole genome level through the selection of entire chromosomal segments (Cao et al., 2016; Zeballos et al., 2016; Hernández Mora et al., 2017). The ideal marker system should be highly polymorphic and evenly distributed across the genome, as well as provide codominant, accurate and reproducible data which can be generated in a high-throughput and cost-effective manner. In association mapping, a dense set of SNP markers covering the entire genome is needed for finding a casual mutation or a SNP that it is in linkage disequilibrium (Flint-Garcia et al., 2005). Association mapping studies requires genotyping platforms capable of producing multi-locus genotypes in a large panel of individuals. Several high-throughput platforms have been developed that allow rapid and simultaneous genotyping of hundreds of thousands of SNPs. The Illumina’s Infinium BeadArray Technology is used for genetic analysis in several crop species, such as barley (Rostoks et al., 2006), soybean (Hyten et al., 2008), and maize (McMullen et al., 2009). Furthermore, high-throughput genotyping arrays using the GoldenGate^®^Assay (Illumina, Inc., San Diego, CA, United States) have previously been used for SNP genotyping in soybean (Hyten et al., 2008), wheat (Akhunov et al., 2009), and maize (Yan et al., 2010). The International Peach SNP Consortium (IPSC) has pursued a genome-scale SNP discovery in peach using next generation sequencing platforms to develop and characterize a high-throughput Illumina Infinium^®^SNP genotyping array platform. The IPSC peach 9 K SNP array v1.0 achieved an average spacing of 26.7 kb between SNPs and distributed over all eight peach chromosomes (Verde et al., 2012). The SNP array has been successfully used in other association mapping studies in peach (Fresnedo-Ramírez et al., 2016; Zeballos et al., 2016; Hernández Mora et al., 2017). The first aim of the present work was to identify genetic regions associated with the most important agronomical and pomological traits in a peach germplasm collection using a medium-size SNP panel covering the peach genome. In previous works, this collection was phenotyped and screened with 40 SSRs markers spanning the peach genome (Font i Forcada, 2012; Font i Forcada et al., 2013, 2014), and significant associations with pomological traits were assessed. Consequently, the second objective for this study was to compare both associations showed in the two different studies and using the same peach germplasm collection.

## Materials and Methods

### Plant Material, Fruit Sampling and Evaluation of Fruit Quality Traits

A germplasm collection of 94 peach and nectarine [*P. persica* (L.) Batsch] cultivars (43 native local Spanish cultivars and 51 modern cultivars mostly from United States, France, Italy, New Zealand, and South Africa) were used in this study, as described by Font i Forcada et al. (2013). Genotypes were grown under typical Mediterranean soil and climate conditions at the Experimental Station of Aula Dei (CSIC) located at Zaragoza (northern of Spain).

The evaluation of the traits was performed over a period of at least 3 years, based on 20 fruits randomly harvested from each cultivar and year at commercial maturity. The entire fruit sample protocol was described by Font i Forcada et al. (2013, 2014). The agronomical and fruit quality traits evaluated were blooming and harvest date (Julian’s day), yield (kg/tree), vigor (TCSA; trunk cross-sectional area; cm^2^), yield efficiency (kg/cm^2^), fruit weight (g), flesh firmness (N), soluble solids content (SSC; Brix), titratable acidity (TA; g malic acid/100 g FW), and ripening index (RI; SSC/TA). Also, biochemical traits as vitamin C (mg of ascorbic acid, AsA per 100 g of FW), anthocyanin content (mg of cyanidin-3-glucoside equivalents, C3GE, per kg of FW), total phenolics (mg of gallic acid, 3,4,5-trihydroxy-benzoic acid, equivalents GAE per 100 g FW), flavonoid content (mg of catechin equivalents per 100 g of FW), relative antioxidant capacity (1,1-diphenyl-2-picrylhydrazyl, DPPH, g of Trolox equivalents per g of FW), and sucrose, glucose, fructose, sorbitol, and total sugars (g/kg FW) were evaluated. All the procedures used in this study were previously described (Font i Forcada et al., 2013, 2014). Phytochemical analyses were performed using a spectrophotometer (Beckman Coulter DU 800) and the individual and total sugars were purified and analyzed using a HPLC (Waters 515, Milford, MA, United States) as described by Font i Forcada et al. (2014).

### DNA Isolation and SNP Analysis

Young leaves were collected from each cultivar, frozen immediately in liquid nitrogen, and stored at -20°C. DNA was isolated using the DNeasy Plant Mini Kit (Qiagen, Dusseldorf, Germany) following the manufacturer’s instructions. DNA concentration and quality was checked using PicoGreen^®^dye and measured in a fluorospectrometer. Then, the 94 cultivars were genotyped using a panel of IPSC 9K peach SNP array v1.0, using the single-base extension assay (Steemers et al., 2006) and Illumina^®^Infinium^®^HD Assay ultra protocol (Illumina, San Diego, CA, United States). The platform was developed by the International Peach SNP Consortium (IPSC) (Verde et al., 2012), which included a set of 8,144 SNPs. The analysis was performed by the ‘Instituto de Investigación Sanitaria (INCLIVA)’ of Valencia (Spain).

The SNP array was developed for use on worldwide breeding germplasm and includes Sanger-based SNPs from genome sequence of ‘Lovell’ generously provided by the International Peach Genome Initiative ^2^.They also include SNPs identified from Illumina 80 bp paired-end genome sequencing of 22 important founder peach accessions (‘Admiral Dewey,’ ‘Slappey,’ ‘Babcock,’ ‘Elberta,’ ‘Carmen,’ ‘Chinese Cling,’ ‘Mayflower,’ ‘Bolinha,’ ‘Yellow St. John,’ ‘J. H. Hale,’ ‘Rio Oso Gem,’ ‘Diamante,’ ‘Dixon,’ ‘Early Crawford,’ ‘Florida Prince,’ ‘Dr. Davis,’ ‘O’Henry,’ ‘Okinawa,’ ‘Nemaguard,’ ‘Lovell,’ ‘Georgia Belle,’ and ‘Oldmixon Free’) and the almond cultivar ‘Nonpareil’ (Verde et al., 2012).

Markers with missing data that are non-polymorphic, redundant, or deviated from the expected segregation proportion were excluded. The workflow for SNP detection, filtering and final choice employed for association analysis are described in Table 1. When markers had the same segregation pattern, only one marker was included to improve computational algorithm efficiency (Van Ooijen, 1992). For the segregation deviation test, a chi-square test was performed with *p* = 0.05 as the threshold.

**Table 1:** Workflow for SNP detection, filtering and final choice employed for association analysis.

	Number of markers	Remaining markers
Detection and validation of Peach 9K array ▶	8,144 SNPs	
After removing monomorphic markers ▶	1,912 SNPs ▶	6,232 polymorphic
After removing markers with gene train score < 0.4 ▶	1,052 SNPs ▶	5,180 SNPs
After removing markers with similar pattern and MAF < 5% ▶	622 SNPs ▶	4,558 SNPs


### Statistical Analysis

#### Population Structure Analysis

The program STRUCTURE v2.3.4 (Pritchard et al., 2000) was used for identification of population structure by clustering individuals into genetically distinguishable groups based on allele frequencies. Structure analysis was performed using 4,558 SNPs on the whole dataset. Analysis was carried out for a range of *K* values from 1 to 10, with 10 runs for each *K*. A burn-in of 5,000 and 50,000 MCMC replications were implemented for each run. The optimal number of *K* clusters was estimated using the Δ*K* parameter of Evanno et al. (2005) in Structure Harvester (Earl and vonHoldt, 2012). Genotypes were subdivided into different populations according to their maximum membership probability among the populations with a threshold of 0.80. Results were displayed graphically in a bar graph/chart.

#### Association Mapping Analysis

Association analysis between DNA marker and agronomical and fruit quality traits was conducted using the software TASSEL v3.0 (Yu and Buckler, 2006). Different models comprising General Linear Model (GLM, Q) and Mixed Linear Model (MLM, Q + K) were selected to calculate *P*-values and to examine association between quality traits and molecular markers. The following models were compared: (a) Naïve-model (GLM without any correction for population structure); (b) Q-model (GLM with Q-matrix as correction for population structure); (c) QK-model (MLM with Q-matrix and K-matrix as correction for population structure and kinship relationships); (d) K-model (MLM with K-matrix as correction for kinship relationships structure). A structured association approach could avoid spurious associations and the results were compared to determine the best model. The significance of marker-traits associations was declared at *P* < 0.05 and a standard correction was performed by applying Bonferroni procedure at *P* < 0.00001097 (Schulze and McMahon, 2002). Alleles with a frequency (MAF) lower than 5% were removed (Wilson et al., 2004). Among the different models, the best model was selected based on the smallest mean square difference (MSD) between the observed and the expected *P*-values, since the random marker *P*-values follow an uniform distribution (Yu et al., 2005). To detect significant markers, the phenotypic variation (*r*^2^) was calculated using a simple regression equation implemented in GLM procedure in TASSEL.

All the bioinformatics analysis were performed on local servers at the UC Davis Genome Center (Davis, CA, United States). Reads were mapped to the reference sequences from the peach genome (Peach v1.0).

#### Power of Detection of QTLs

Computer simulations were used to determine the power of detection of QTLs with the current population and marker set. For this analysis, a modified version of the phenosym R program (Maruki and Lynch, 2015) was used to enable simulations across a complete panel of heritability values and QTL sizes. The simulations were performed iteratively in combination of heritability values and QTL sizes from 0 to 0.9 in 0.1 increments (in total 100 simulations) with 1000 iterations per simulation to determine the power of detection. After identifying the detection power of the population additional simulations were performed with varying distance between the predicted QTL and the SNP to study LD values.

## Results

### Genotyping of Peach and Nectarine Cultivars Using SNP Array

The Tassel pipeline initially identified 8,144 SNP loci distributed across all major scaffolds of the peach genome. Data were filtered to remove SNPs with low read support. Data for individual SNPs were retained only if they possessed polymorphic markers (6,232 SNPs remained), and with the gene train score lower than 0.4 (5,180 SNPs remained). In addition, data were filtered after removing the markers with similar pattern and with minor allele frequency (lower than 5%). After discovery and amplification on the Infinium HD BeadChips Illumina, 4,558 high quality SNPs remained for the final analysis of genetic structure and association studies, as summarized in Table 1. The final number of SNPs was distributed as follow: 653 SNPs on the scaffold 1; 735 on the scaffold 2; 499 on the scaffold 3; 842 on the scaffold 4; 348 on the scaffold 5; 576 on the scaffold 6; 410 on the scaffold 7; and 495 on the scaffold 8.

### Genetic Structure Among Peach and Nectarine Cultivars

**FIGURE 1:**
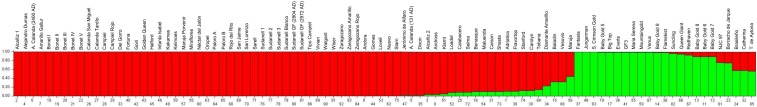
Population structure at *K* = 2 for the 94 peach/nectarine cultivars analyzed in this study.

The population genetic structure determination among 94 peach and nectarine cultivars using genotyping data of genome-wide SNPs markers classified the cultivars in two distinct subpopulations at a *K* value of 2 (Figure 1). The main group (subpopulation A) was formed by 67 cultivars, with 38 Spanish local cultivars and 29 modern cultivars. The second group (subpopulation B) was formed by 18 modern cultivars, with USA as the origin of most of them. Six out seven nectarines analyzed in this study were grouped in subpopulation B. By considering admixed cultivars (seven genotypes), most of them have a European origin (Spain and Italy) and there is not available information about their parentage. Cultivars with known parentage information, such as ‘Andora’ and ‘Carolyn,’ which come from the ‘Libbee’ x ‘Lovell’ cross, were grouped in population A, while ‘Baby Gold 5,’ ‘Baby Gold 8,’ ‘Baby Gold 9,’ ‘Mountain Gold’ and ‘Suncling’ (which share ‘PI35201’ as the maternal genotype), were grouped in a same subpopulation (subpopulation B).

### Population Structure Analysis

Association analysis was performed in the germplasm collection of 94 peach and nectarine cultivars. Marker-trait associations were obtained for agronomical, fruit quality and biochemical traits. The mean phenotypic data obtained during 3 years of evaluation (described in Font i Forcada et al., 2014) was used to test the association analysis with the 4,558 polymorphic SNPs markers.

We tested four models in TASSEL software to determine associations and to account for the influence of population structure by comparing their ability to reduce the inflation of false positive associations. The *P*-values were plotted in a cumulative fashion for each model and the distribution examined. According to Stich et al. (2008) the distribution of *P*-values ideally should follow an uniform distribution with less deviation from the expected *P*-values.

The association analysis using the GLM approach (being the naïve model) and Q-model, detected a large number of associations between the markers and phenotypes which after Bonferroni correction for multiple testing of accessions, reduced down the number of associations. The Q-model showed 66 associations for the evaluated traits. It appears that these models may not have accounted for the heterogeneity of the genetic background, which may have resulted in false positive associations.

### Identification of SNPs Associated With Fruit Quality Traits

**FIGURE 2:**
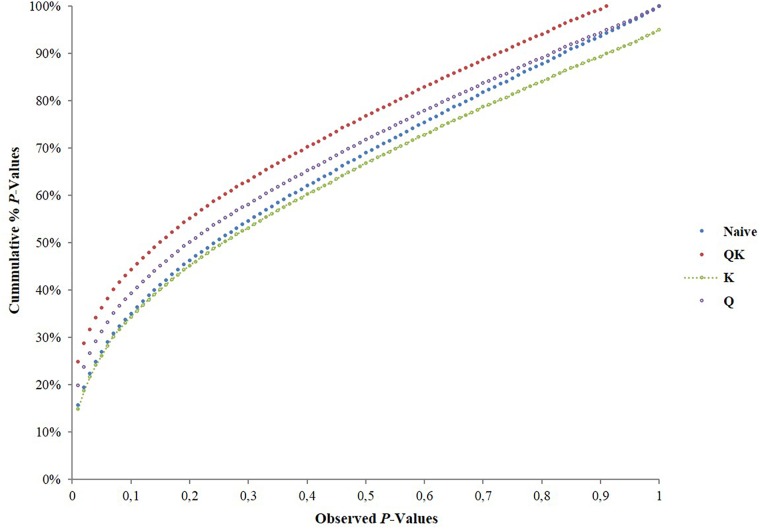
Comparison of four genomes wide association study model: naïve-model (GLM without any correction for population structure); Q-model (GLM with Q-matrix as correction for population structure); QK-model (MLM with Q-matrix and K-matrix as correction for population structure and kinship relationships); and K-model (MLM with K-matrix as correction for kinship relationships structure). Cumulative distribution of *P*-values was computed from the DNA markers and phenotypes for the different association models.

**FIGURE 3:**
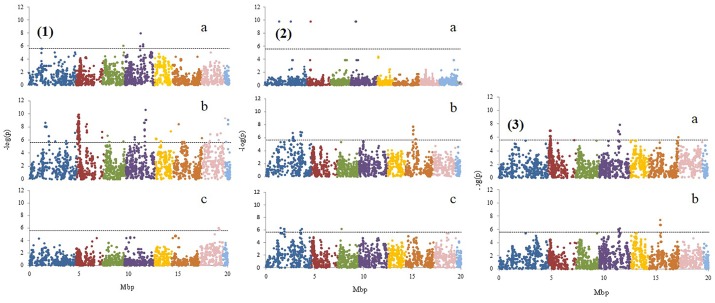
Genome scan showing –log (*p*) value for marker associations (K-model) with **1**: (a) blooming date, (b) harvest date, and (c) ripening index; **2**: (a) anthocyanins, (b) flavonoids, and (c) relative antioxidant capacity; **3**: (a) sorbitol and (b) total sugars. The different colors represent the different linkage groups from 1 to 8.

The K-model and QK-model showed good fit for the *P*-values (*P* < 0.00001298), while the other models were characterized by the excess of small *P*-values (abundance of spurious associations; Figure 2). These two later models showed high uniform distribution of *P*-values. Taking into account the performance of the different models, only results from the K-model will be presented and discussed here since this appeared to have controlled population structure and kinship relationships better. A total of 347 associations (Table 2, Figure 3, and Supplementary File 1) in different scaffolds were found with blooming date (scaffolds: 1, 3, and 4), harvest date (scaffolds: 1, 2, 3, 4, 5, 6, 7, and 8), ripening index (scaffold: 7), anthocyanins (scaffolds: 1, 2, and 4), flavonoids (scaffolds: 1 and 6), RAC (scaffolds: 1 and 3), sorbitol (scaffolds: 2, 4, 6, and 8), and total sugars (scaffolds: 4, 6, and 8). Only harvest date showed associations in all scaffolds. The maximum number of associations was found in scaffold 2 (164), followed by scaffold 1 (55) and scaffold 4 (42), and the minimum number of associations (5) were found on scaffold 3. Only eight out the seventeen quality traits used, showed associations with the SNPs based on the 9k SNP array (v1.0). Figure 3 represents the associations by trait and by scaffold. It is interesting to note that some associations were also observed in the same regions where several QTLs using SSR markers had previously been identified (Tables 2, 3). The percentage of phenotypic variation detected by the associations ranged between 21.62 and 87.52%. We have detected four SNPs in agreement with the position of the SSRs markers reported by Font i Forcada et al. (2013) (Table 3). These markers are: CPPCT028 SSR marker associated with harvest date and sorbitol linkage group (LG) 4 and near to SNP_IGA_450711 in the position 20,165.259 bp; UDP96-003 SSR marker associated with anthocyanin content (LG4) and near to SNP_IGA_395202 in the position 6,168.570 bp; the CPPCT030 associated with harvest date and sorbitol (LG6) and near to SNP_IGA_700469 in the position 28,045.174 bp; and the UDP96-001 SSR marker associated with harvest date and flavonoids (LG6) and near to SNP_IGA_630302 in the position 8,238.299 bp.^3^ Comparing both studies, with SSRs (Font i Forcada et al., 2013) and SNPs markers, significant associations were also found with the same traits when using 40 SSRs markers and covering all the peach genome. The previous work showed association between SSR markers and harvest date in LG4 and LG6, RI in LG2, LG5 and LG6, flavonoids in LG2 and LG4, anthocyanins in LG2, LG3, LG4, LG5, LG6, and LG8, RAC in LG4 and LG6, sorbitol in LG4 and LG5, and total sugars in LG2 and LG4. The position on the physical map for the UDP98-410, endoPG1 and BPPCT015 SSR markers, where several associations were found, are unknown. The four transcripts corresponding to the SNP_IGA_450711, SNP_IGA_395202, SNP_IGA_700469, and SNP_IGA_630302 were identified as ppa023762m (Prupe.4G257400), ppa002262m (Prupe.4G114800), ppa006828m (Prupe.6G350900), and ppa019744m (Prupe.6G114400), respectively. Among them, the common associations found in both studies were obtained with the UDP98-410, BPPCT015, endoPG1, CPPCT028, UDP96-003, CPPCT030, and UDP96-001 SSR markers associated with anthocyanin content, harvest date, total sugars, and sorbitol content.

**Table 2:** Short list of the SNPs associated with different pomological traits, closest markers at the flanking map position, and their *p*-value.

Scaffold	Closest marker at the flanking map position	Trait associated	No. of associations	Flanking interval length (bp)	*p*-value
Pp01	SNP_IGA_37843	Blooming date	1	12,641.440	^∗∗^
Pp01	SNP_IGA_46754-132155	Harvest date	23	14,980.305–44,936.042	^∗∗∗^
Pp01	SNP_IGA_53531-96167	Anthocyanins	4	15,750.283–28,550.473	^∗∗∗^
Pp01	SNP_IGA_82861-112690	Flavonoids	18	23,722.082–36,758.815	^∗∗∗^
Pp01	SNP_IGA_48586-112690	RAC	9	15,234.386–36,758.815	^∗∗∗^
Pp02	SNP_IGA_137253-287700	Harvest date	144	461.255–25,228.844	^∗∗∗^
Pp02	SNP_IGA_181444	Anthocyanins	1	3,800.271	^∗∗∗^
Pp02	SNP_IGA_152976-287700	Sorbitol	19	1,761.256–25,228.844	^∗∗∗^
Pp03	SNP_IGA_365780	Blooming date	1	20,635.992	^∗∗∗^
Pp03	SNP_IGA_303724-363719	Harvest date	3	4,002.228–19,759.990	^∗∗∗^
Pp03	SNP_IGA_303724	RAC	1	4,002.228	^∗∗∗^
Pp04	SNP_IGA_430583-441904	Blooming date	6	15,574.015–18,522.596	^∗∗^
Pp04	SNP_IGA_403353-450711	^a^Harvest date	15	8,996.802–20,165.259	^∗∗∗^
Pp04	SNP_IGA_392956-395202	Anthocyanins	4	5,689.470–6,168.570	^∗∗∗^
Pp04	SNP_IGA_442063-450711	Sorbitol	10	18,548.028–20,165.259	^∗∗∗^
Pp04	SNP_IGA_442063-449112	^a^Total sugars	7	18,548.028–19,905.501	^∗∗∗^
Pp05	SNP_IGA_543247-600691	Harvest date	13	276.220–14,995.466	^∗∗∗^
Pp06	SNP_IGA_619807-700469	Harvest date	4	4,759.496–28,045.174	^∗∗∗^
Pp06	SNP_IGA_628833-638859	Flavonoids	15	7,901.344–11,016.846	^∗∗∗^
Pp06	SNP_IGA_700469	Sorbitol	1	28,045.174	^∗∗∗^
Pp06	SNP_IGA_636024-637355	^a^Total sugars	5	10,460.202–10,606.410	^∗∗∗^
Pp07	SNP_IGA_746619-792898	Harvest date	9	7,470.226–22,673.209	^∗∗∗^
Pp07	SNP_IGA_784373-786935	RI	10	18,510.773–19,542.449	^∗∗∗^
Pp08	SNP_IGA_797680-879224	Harvest date	17	1,271.540–18,309.578	^∗∗∗^
Pp08	SNP_IGA_878717-879224	Sorbitol	5	18,085.149–18,309.578	^∗∗∗^
Pp08	SNP_IGA_870629-879224	Total sugars	2	15,787.171–18,309.578	^∗∗^


*For multiple testing of genotypes, Bonferroni correction (Schulze and McMahon, 2002) was applied. For the *p*-value: ^∗∗^ refers to *p* ≤ 0.000001–0.0000001; and ^∗∗∗^ refers to *p* ≤ 0.0000001–0.00000001 (K-model). ^*a*^Associations observed in the same regions where QTLs have previously been identified (Etienne et al., 2002; Cantín et al., 2010a; Eduardo et al., 2011).*

**Table 3:** List of SSR markers, linkage group (LG), scaffolds, SNP location, SNP marker, and traits associated comparing both studies, with SSRs (Font i Forcada et al., 2013) and SNP markers.

SSR marker	LG	Scaffold	SNP Location	SNP marker	Traits associated
UDP98-410	2	Unknown	Unknown	Unknown	Anthocyanin
BPPCT015	4	Unknown	Unknown	Unknown	Harvest date
BPPCT015	4	Unknown	Unknown	Unknown	^a^Total sugars
BPPCT015	4	Unknown	Unknown	Unknown	Sorbitol
endoPG1	4	Unknown	Unknown	Unknown	^a^Harvest date, ^a^Total sugars
CPPCT028	4	4	20,165.259	SNP_IGA_450711	Harvest date, Sorbitol
UDP96-003	4	4	6,168.570	SNP_IGA_395202	Anthocyanin
CPPCT030	6	6	28,045.174	SNP_IGA_700469	Harvest date, Sorbitol
UDP96-001	6	6	8,238.299	SNP_IGA_630302	Harvest date, Flavonoid


*^a^Associations observed in the same regions where QTLs had previously been identified (Etienne et al., 2002; Cantín et al., 2010a; Eduardo et al., 2011).*

**FIGURE 4:**
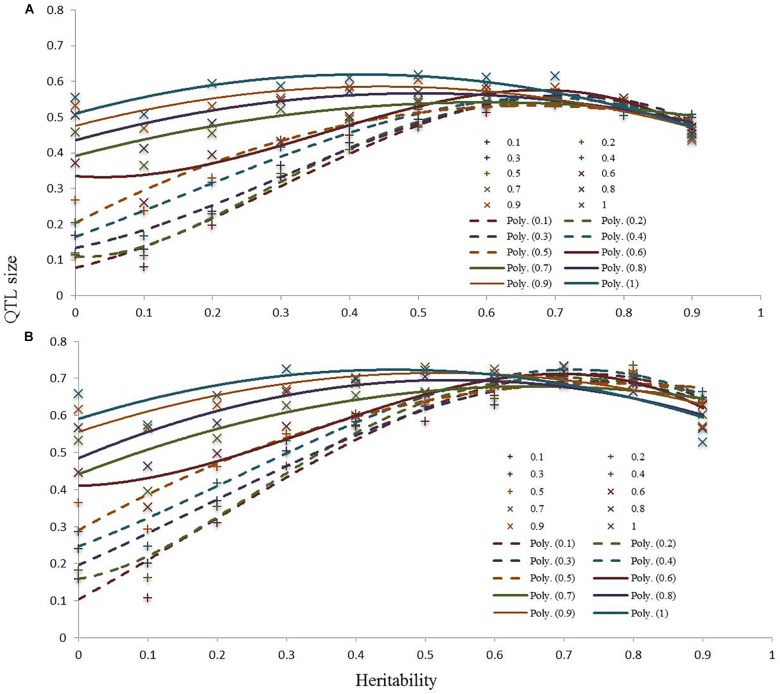
Computer simulations at 250 kb **(A)** and 500 kb **(B)** to determine the power of detection of QTLs.

The power of detection of QTLs showed that, as expected, the effect of the QTL size is inversely proportional to the heritability. We can see, as shown in Figure 4 for high heritability values (over 0.5), the prediction power tends to be constant across the QTL sizes. For the lower heritability values, a linear correlation can be seen between QTL size and prediction power.

## Discussion

### Population Structure

By using the STRUCTURE program to analyze the population structure of 94 peach and nectarine cultivars used in this study, the maximum Δ*K*-value was observed for *K* = 2. This result suggests that cultivars were grouped in two main subpopulations, plus a group of admixed cultivars. Peaches and nectarines were separated in two different subpopulations and cultivars with known parentage information were grouped with their parental genotypes. In a previous study (Font i Forcada et al., 2013) for the same group of cultivars, but using SSRs markers for association mapping in peach/nectarine cultivars, the maximum Δ*K*-value was observed for *K* = 3. This difference between both studies could be generated because of the different markers used in the previous work (SSRs) and those used in the present analysis (SNPs). Although SNPs are less polymorphic than SSR markers because of their biallelic nature, they easily compensate this drawback by being abundant, ubiquitous, and amenable to high- and ultra-high-throughput automation (Mammadov et al., 2012). In addition, the availability and stability of SNPs in comparison to SSRs provide better prospects for cultivar identification and assessment of genetic diversity (Fernández i Martí et al., 2012). In the present study, the main subpopulation grouped local Spanish and foreign modern cultivars, the latter from international breeding programs that are maintained at the Experimental Station of Aula Dei, Spain. It is known that after the dispersion of peach from China through Persia to Europe, a much more recent (16th-19th century) introduction of peach to the United States is represented by a few cultivars that have subsequently served as the genetic foundation of the modern breeding germplasm (Scorza et al., 1985). It is probable that several of the modern cultivars used in this study, without information about their parentage, have local European or Spanish cultivars as their parental genotypes and this could be the reason because an important group of local Spanish cultivars grouped together with modern cultivars, most of them from USA breeding programs.

### Association Analysis

Association mapping is increasingly being utilized to detect marker-QTL linkage associations using plant material developed in breeding programs (Zeballos et al., 2016). Association mapping could be a more practical approach for cultivar development, considering that markers linked to major QTLs may immediately be utilized in marker assisted selection, once new QTLs are identified (Oraguzie et al., 2007).

High-density SNP phenotyping arrays are powerful tools for studying genomic patterns of diversity and marker-trait associations in mapping experiments (Muranty et al., 2014). The IPSC 9k SNP array v1.0 (Verde et al., 2012) consisted of 8,144 working SNPs, among which 80% were polymorphic across 94 cultivars of peach and 5% failed. In our study, 4,558 high quality SNPs remained for the final analysis of association studies (55.96%). These percentage of polymorphic SNPs is slightly lower comparing the study reported by Verde et al. (2012) for SNPs with MAF >0.1 (71.4%) and by Lambert et al. (2016) with MAF >0.1 (64.5%) suggesting that only 2/3 of the SNPs distributed in the 9K array v1.0 were finally useful.

Checking the literature, it exists more information in *Prunus* species on QTLs based on agronomical, morphological or basic fruit quality traits than QTLs based on biochemical traits. Important QTLs that control fruit quality traits have been found for organic acid content, fruit weight, sub-acidity fruit (D), and blooming and harvest dates (Etienne et al., 2002); blooming and ripening dates (Eduardo et al., 2011; Zeballos et al., 2016) and chilling injury susceptibility (Cantín et al., 2010a). However, the availability of SNP genotyping resources has assisted in fine mapping of peach (Zhebentyayeva et al., 2014). Several QTLs that control traits such as chilling and heat requirements (Romeu et al., 2014), maturity date or other pomological traits, such as fruit weight, soluble solid content, or pH (Eduardo et al., 2013; Fresnedo-Ramírez et al., 2015; Zeballos et al., 2016), have been mapped. Different studies for QTL identification in one apricot population (Salazar et al., 2016) identified major QTLs for ripening date (or harvest date) in LG4, showing that the nearest marker to major ripening date were the UDP96-003 marker. These results are in agreement with those found in our study. They showed the SNP_IGA_450711 associated with harvest date is in LG4 (Table 2 and Supplementary File 1). In addition, the marker closest to the SNP_IGA_395202 (Table 3) was the SSR marker UDP96-003 but associated with anthocyanin content instead of ripening date. However, more studies are needed in this area to facilitate QTL co-localization and/or synteny analysis among the *Prunus* species, with a view to undergoing candidate gene identification and fine mapping.

This genome-wide association study based on a panel of 94 peach cultivars and the ISPC 9K SNP array has identified a set of SNPs associated with different traits. Comparing this study with the previous one using SSRs (Font i Forcada et al., 2013), similar significant associations were found between the fruit traits and the different markers covering all the peach genome. The common associations found in both studies were obtained with the UDP98-410, BPPCT015, CPPCT028, UDP96-003, CPPCT030, endoPG1 and UDP96-001 SSR markers with harvest date, anthocyanin, sorbitol and total sugars. A different association mapping study with SSRs and 104 peach landraces from China showed the CPPCT005 marker in LG4 and the UDP98-407 marker in LG6 associated with blooming date (Cao et al., 2012), in good agreement with our study. Unfortunately, the positions of these markers on the physical map are unknown ^4^. Another study with SSRs (Fan et al., 2010) also found QTLs for blooming date in LG1, but the positions of the markers on the physical map are also unknown. It is interesting to note that, we have found associations between SNPs markers and blooming date in scaffolds 1, 3, and 4. In contrast, the study with SSRs (Font i Forcada et al., 2013) did not show any association with blooming date. These results could be very useful because many of the associated markers (both, SSRs and SNPs) were located in common regions where major genes or QTLs for fruit quality traits have already been detected and have been previously identified and mapped on the *Prunus* reference map (Arús et al., 2012). Guan et al. (2015) reported that several QTLs for fructose, glucose, sucrose, and sorbitol were clustered. This interesting clustering of QTLs may be caused by the tight linkage among separate controlling genes or by the pleiotropic effect of a single gene. Eduardo et al. (2011) found a cluster containing QTLs for maturity date, fructose content, sucrose content, titrable acidity, and pH, also found in Dirlewanger et al. (1999) near to marker BPPCT15 on the G4. In the same region, Quilot et al. (2004) mapped a cluster of QTLs associated with maturity date and content of sorbitol, fructose, maltose, citric acid, and quinic acid. In our case, by analyzing individuals from a peach germplasm collection using association mapping, the significant associations found between the markers BPPCT015 and endoPG1 in LG4 with harvest date (highly correlated to maturity date), total sugars and sorbitol suggest a pleiotropic effect as previously reported by using bi-parental populations and QTLs identification. It will be interesting to decipher the biological processes underlying this strong pleiotropic effect in order to allow the identification of QTLs in future works. Pomological traits associated with genotypic traits seem consistent with previous studies where QTLs were mapped on LG1 for blooming date (Fan et al., 2010; Zeballos et al., 2016), and on LG4 with harvest date (Cantín et al., 2010a; Eduardo et al., 2011; Arús et al., 2012; Zeballos et al., 2016) and sugars content (Etienne et al., 2002; Arús et al., 2012; Zeballos et al., 2016). However, some associations were not consistent with the results of other published linkage analyses. These discrepancies in marker-locus-trait associations between the different studies could be attributed to number of marker loci used. Although the small sample size can cause a loss of power or provide associations that are not of general applicability (Hackshaw, 2008), they are valid for the set of genotypes used in the present study. In fact, this research includes many peach cultivars relevant to the peach industry in addition to material developed in different international breeding programs. Nevertheless, a larger confirmatory study should be carried out in the future.

#### Candidate Genes

Our aim was to relate these associations to structural genes using a candidate gene/QTL approach. We have detected two SNPs (SNP_IGA_449112 and SNP_IGA_450711) in the promoter of the candidate gene ppa025636m that was associated with total sugars content and sorbitol, respectively, in LG4 and in the interval comprised between 18,085 to 20,165.259 bp. Also, the SNP_IGA_450711 position, is in agreement with the position of CPPCT028 SSR marker found in the study of Font i Forcada et al. (2013). Lambert et al. (2016) reported that fruit stone adhesion and flesh texture (F-M) co-segregated in three mapping progenies in LG4 with an interval comprised between 18,593.828 and 20,176.048 bp, similar to our results, and also, the flanking map position included the SNP_IGA_449112 and the SNP_IGA_450711, according with our results.

We have founded three other SNPs associations in common with both studies, using SSRs (Font i Forcada et al., 2013) and SNPs (Table 3 and Supplementary File 2). Firstly, the UDP96-003 SSR marker was associated with anthocyanin content and very close to SNP_IGA_395202 (candidate gene ppb015985m). This gene encodes a cyclic GMP-activated non-selective cation channel in the plasma membrane of guard cells ^5^. Second one, the lower region of the LG6 and close to marker CPPCT030 and one gene, ppa006828m, were identified very close to the position of SNP_IGA_700469, and associated with harvest date and sorbitol content. It may be responsible for the protein involved in peroxisome biogenesis^5^. This gene is expressed during the flowering stage, the mature plant embryo stage, and the petal differentiation and expansion stage. This is a fatty acid oxidation process that results in the complete oxidation of a long-chain fatty acid and a fatty acid beta-oxidation begins with the addition of coenzyme A to a fatty acid, and occurs by successive cycles of reactions during each of which the fatty acid is shortened by a two-carbon fragment removed as acetyl coenzyme A^5^. This candidate gene is also in agreement with that proposed on almonds trees (Font i Forcada et al., 2012, 2015), including Acyl-CoA, controlling the synthesis of long-chain saturated fatty acids. In the present work, it appears to be within the interval of the association found for the ppa006828m gene. Similar function was found with the gene ppa019942m (Supplementary File 2), associated with harvest date. The UDP96-001 marker was associated with harvest date and it is close to the SNP_IGA_630302 (candidate gene ppa024155m). This gene seems to be expressed during the L mature pollen stage, M germinated pollen stage and flowering stage^5^.

In our study, four transcription factors related to anthocyanin biosynthesis were found. In plants, the R gene product Lc, which is involved in the control of anthocyanin synthesis in maize, was the first plant protein reported to possess a bHLH motif (Ludwig et al., 1989). The bHLH is involved in the regulation of the anthocyanin pathway and it has been identified in peach (Ravaglia et al., 2013) and some other fruit species (Hichri et al., 2010; Xie et al., 2012). Here, we identified ppa022224m and ppa021918m (LG1), ppa019868m (LG2), and ppa019552m (LG7) genes through a phylogenetic tree of *Arabidopsis* and peach bHLHbZIP family members (Supplementary File 2). Among them, ppa022224m and ppa021918 genes were highly correlated with the flavonoid and RAC values. These traits are also highly correlated with the anthocyanin trait as described by Font i Forcada et al. (2013, 2014). Similar results were found in other peach studies (Zhao et al., 2017).

Finally, the candidate gene, ppa021329m, on chromosome 6 (Supplementary File 2), shows similarity to lycopene cyclase at3g10203 in the *Arabidopsis thaliana* genome and associated with harvest date and flavonoids content. Lycopene cyclases (LYCs) play a role in the biosynthesis of lutein, which is a member of the carotenoids pathway (Giuliano et al., 2008). This candidate gene may be associated with the yellow carotenoid pigmentation in peach and correlated with flavonoid content. Although the flesh coloration in peach is determined by the locus Y (LG1), major QTLs for skin color were also found (Eduardo et al., 2011) in different chromosomes (LG4, LG6 and LG7).

#### Datasets Are Available on Request

The raw data supporting the conclusions of this manuscript will be made available by the authors, without undue reservation, to any qualified researcher.

## Conclusion

The present study, using the IPSC peach SNP array v1.0, allowed finding associations with agronomical and pomological traits in a wide peach germplasm collection. Due to the density of markers, this work confirm and reinforce results found with different markers type (SSRs and SNPs) to know if there is any impact on the results of GWA mapping in peach. Peach association mapping is an alternative to QTL mapping based on crosses between different cultivars because of the multiple advantage compared to bi-parental populations. Finding specific regions of the genome will provide further information searching for the genes involved in peach fruit quality, and to identify new candidate genes. Additionally, this work provide promising results concerning association mapping with pomological traits that could be applied in other *Prunus* species because of synteny inside the Rosaceae family, and it would be very useful to make predictions of genetic progress in breeding programs.

## Author Contributions

CFiF and MM designed the study and the experiments. MM provided the material from the peach germplasm collection. CFiF, VG, and SC-W performed the statistical and biochemical analysis. CFiF, VG, and MM discussed the data, wrote and reviewed the manuscript. All authors have read and approved the final manuscript.

## Conflict of Interest Statement

The authors declare that the research was conducted in the absence of any commercial or financial relationships that could be construed as a potential conflict of interest.

## Footnotes

1 http://faostat.fao.org/

2 www.rosaceae.org/peach/genome

3 https://www.rosaceae.org

4 www.rosaceae.org

5 www.arabidopsis.org
