# Supplementary material for: Association Mapping Analysis for Fruit Quality Traits in Prunus persica Using SNP Markers
Source: Front Plant Sci. 2019 Jan 17;9:2005. doi: 10.3389/fpls.2018.02005 (PMC6344403; doi:10.3389/fpls.2018.02005)
Supplement: Supplementary file 1 [file Table_1.DOCX]

Supplementary file 1. Complete list of the 347 SNPs associated with different pomological traits, closest markers at the flanking map position, and their p-value.

| **Trait associated** | **Closest marker at the flanking map position** | **Scaffold** | **Flanking interval length (bp)** | ***p*-value** |
| --- | --- | --- | --- | --- |
| Blooming date | SNP_IGA_37843 | Pp01 | 12641440 | 2.5655E-06 |
| Harvest date | SNP_IGA_46754 | Pp01 | 14980305 | 8.8E-09 |
| Harvest date | SNP_IGA_48586 | Pp01 | 15234386 | 2.3E-09 |
| Harvest date | SNP_IGA_55903 | Pp01 | 15890555 | 8.8E-09 |
| Harvest date | SNP_IGA_56163 | Pp01 | 15907025 | 8.8E-09 |
| Harvest date | SNP_IGA_56198 | Pp01 | 15908997 | 8.8E-09 |
| Harvest date | SNP_IGA_57051 | Pp01 | 16186175 | 1.14E-08 |
| Harvest date | SNP_IGA_57241 | Pp01 | 16202261 | 1.14E-08 |
| Harvest date | SNP_IGA_57544 | Pp01 | 16389065 | 8.8E-09 |
| Harvest date | SNP_IGA_57793 | Pp01 | 16426046 | 8.8E-09 |
| Harvest date | SNP_IGA_63566 | Pp01 | 18553050 | 1.7881E-06 |
| Harvest date | SNP_IGA_63603 | Pp01 | 18560713 | 1.7881E-06 |
| Harvest date | SNP_IGA_63638 | Pp01 | 18571836 | 1.7881E-06 |
| Harvest date | SNP_IGA_63776 | Pp01 | 18603486 | 1.7881E-06 |
| Harvest date | SNP_IGA_63825 | Pp01 | 18621833 | 2.418E-07 |
| Harvest date | SNP_IGA_63833 | Pp01 | 18622159 | 2.418E-07 |
| Harvest date | SNP_IGA_63847 | Pp01 | 18623934 | 2.418E-07 |
| Harvest date | SNP_IGA_64918 | Pp01 | 18762086 | 2.418E-07 |
| Harvest date | SNP_IGA_65002 | Pp01 | 18809420 | 2.418E-07 |
| Harvest date | SNP_IGA_65286 | Pp01 | 18911503 | 1.7881E-06 |
| Harvest date | SNP_IGA_86245 | Pp01 | 25343182 | 1.7344E-06 |
| Harvest date | SNP_IGA_86252 | Pp01 | 25343795 | 1.7344E-06 |
| Harvest date | SNP_IGA_111017 | Pp01 | 35798416 | 1.4258E-06 |
| Harvest date | SNP_IGA_132155 | Pp01 | 44936042 | 7.69E-08 |
| Anthocyanins | SNP_IGA_53531 | Pp01 | 15750283 | 2E-10 |
| Anthocyanins | SNP_IGA_15750387 | Pp01 | 15750387 | 2E-10 |
| Anthocyanins | SNP_IGA_54637 | Pp01 | 15844751 | 2E-10 |
| Anthocyanins | SNP_IGA_96167 | Pp01 | 28550473 | 2E-10 |
| Flavonoids | SNP_IGA_82861 | Pp01 | 23722082 | 2.4454E-06 |
| Flavonoids | SNP_IGA_93589 | Pp01 | 27651740 | 1.972E-07 |
| Flavonoids | SNP_IGA_93646 | Pp01 | 27677094 | 1.2306E-06 |
| Flavonoids | SNP_IGA_93768 | Pp01 | 27715921 | 1.2306E-06 |
| Flavonoids | SNP_IGA_94024 | Pp01 | 27815137 | 7.409E-07 |
| Flavonoids | SNP_IGA_94057 | Pp01 | 27821450 | 7.409E-07 |
| Flavonoids | SNP_IGA_95319 | Pp01 | 28342277 | 2.0014E-06 |
| Flavonoids | SNP_IGA_96046 | Pp01 | 28534000 | 2.0014E-06 |
| Flavonoids | SNP_IGA_96144 | Pp01 | 28547153 | 2.5583E-06 |
| Flavonoids | SNP_IGA_96544 | Pp01 | 28663689 | 2.5583E-06 |
| Flavonoids | SNP_IGA_96555 | Pp01 | 28677236 | 2.5583E-06 |
| Flavonoids | SNP_IGA_110227 | Pp01 | 35577807 | 4.035E-07 |
| Flavonoids | SNP_IGA_110232 | Pp01 | 35578084 | 4.035E-07 |
| Flavonoids | SNP_IGA_110413 | Pp01 | 35617714 | 1.371E-07 |
| Flavonoids | SNP_IGA_111017 | Pp01 | 35798416 | 0.000000361 |
| Flavonoids | SNP_IGA_111259 | Pp01 | 36067680 | 5.569E-07 |
| Flavonoids | SNP_IGA_111329 | Pp01 | 36098186 | 5.569E-07 |
| Flavonoids | SNP_IGA_112690 | Pp01 | 36758815 | 0.000000168 |
| Relative Antioxidant Capacity | SNP_IGA_48586 | Pp01 | 15234386 | 4.932E-07 |
| Relative Antioxidant Capacity | SNP_IGA_63825 | Pp01 | 18621833 | 7.245E-07 |
| Relative Antioxidant Capacity | SNP_IGA_63833 | Pp01 | 18622159 | 7.245E-07 |
| Relative Antioxidant Capacity | SNP_IGA_63847 | Pp01 | 18623934 | 7.245E-07 |
| Relative Antioxidant Capacity | SNP_IGA_64918 | Pp01 | 18762086 | 7.245E-07 |
| Relative Antioxidant Capacity | SNP_IGA_65002 | Pp01 | 18809420 | 7.245E-07 |
| Relative Antioxidant Capacity | SNP_IGA_65816 | Pp01 | 19085285 | 1.7499E-06 |
| Relative Antioxidant Capacity | SNP_IGA_110413 | Pp01 | 35617714 | 1.1894E-06 |
| Relative Antioxidant Capacity | SNP_IGA_112690 | Pp01 | 36758815 | 7.554E-07 |
| Harvest date | SNP_IGA_137253 | Pp02 | 461255 | 6.505E-07 |
| Harvest date | SNP_IGA_137307 | Pp02 | 474944 | 4.97E-08 |
| Harvest date | SNP_IGA_137745 | Pp02 | 512699 | 6.505E-07 |
| Harvest date | SNP_IGA_137839 | Pp02 | 529491 | 4.3E-09 |
| Harvest date | SNP_IGA_138882 | Pp02 | 654242 | 4.3E-09 |
| Harvest date | SNP_IGA_139270 | Pp02 | 692014 | 6.795E-07 |
| Harvest date | SNP_IGA_139612 | Pp02 | 713093 | 3.72E-08 |
| Harvest date | SNP_IGA_140089 | Pp02 | 732572 | 3.632E-07 |
| Harvest date | SNP_IGA_140105 | Pp02 | 733147 | 3.72E-08 |
| Harvest date | SNP_IGA_140371 | Pp02 | 745074 | 3.72E-08 |
| Harvest date | SNP_IGA_140441 | Pp02 | 748755 | 6.795E-07 |
| Harvest date | SNP_IGA_140557 | Pp02 | 754089 | 3.7E-09 |
| Harvest date | SNP_IGA_140573 | Pp02 | 754603 | 3.72E-08 |
| Harvest date | SNP_IGA_140599 | Pp02 | 755371 | 3.7E-09 |
| Harvest date | SNP_IGA_140823 | Pp02 | 775785 | 0.000000004 |
| Harvest date | SNP_IGA_140857 | Pp02 | 776722 | 0.000000004 |
| Harvest date | SNP_IGA_140864 | Pp02 | 776885 | 6.01E-08 |
| Harvest date | SNP_IGA_140922 | Pp02 | 778085 | 0.000000004 |
| Harvest date | SNP_IGA_140933 | Pp02 | 778300 | 6.01E-08 |
| Harvest date | SNP_IGA_140938 | Pp02 | 778744 | 3.7E-09 |
| Harvest date | SNP_IGA_140951 | Pp02 | 779117 | 0.000000004 |
| Harvest date | SNP_IGA_140965 | Pp02 | 779808 | 0.000000004 |
| Harvest date | SNP_IGA_140990 | Pp02 | 780479 | 3.7E-09 |
| Harvest date | SNP_IGA_141600 | Pp02 | 879987 | 2.6E-09 |
| Harvest date | SNP_IGA_141607 | Pp02 | 880882 | 2.6E-09 |
| Harvest date | SNP_IGA_141612 | Pp02 | 881016 | 2.6E-09 |
| Harvest date | SNP_IGA_141624 | Pp02 | 881365 | 3E-10 |
| Harvest date | SNP_IGA_141858 | Pp02 | 911846 | 4.242E-07 |
| Harvest date | SNP_IGA_141868 | Pp02 | 913067 | 6.834E-07 |
| Harvest date | SNP_IGA_142214 | Pp02 | 941180 | 6.834E-07 |
| Harvest date | SNP_IGA_142231 | Pp02 | 942619 | 5.48E-08 |
| Harvest date | SNP_IGA_142473 | Pp02 | 961984 | 3.145E-07 |
| Harvest date | SNP_IGA_142518 | Pp02 | 964244 | 3.7E-09 |
| Harvest date | SNP_IGA_143002 | Pp02 | 1011959 | 5.48E-08 |
| Harvest date | SNP_IGA_143150 | Pp02 | 1023786 | 4.899E-07 |
| Harvest date | SNP_IGA_143346 | Pp02 | 1034154 | 2.139E-07 |
| Harvest date | SNP_IGA_143360 | Pp02 | 1035269 | 2.139E-07 |
| Harvest date | SNP_IGA_143796 | Pp02 | 1052865 | 3.7E-09 |
| Harvest date | SNP_IGA_143809 | Pp02 | 1053489 | 0.000000004 |
| Harvest date | SNP_IGA_143907 | Pp02 | 1057628 | 2.93E-08 |
| Harvest date | SNP_IGA_143917 | Pp02 | 1057806 | 0.000000004 |
| Harvest date | SNP_IGA_143925 | Pp02 | 1058060 | 0.000000004 |
| Harvest date | SNP_IGA_144878 | Pp02 | 1116293 | 2.139E-07 |
| Harvest date | SNP_IGA_144902 | Pp02 | 1117840 | 4.605E-07 |
| Harvest date | SNP_IGA_144913 | Pp02 | 1118746 | 2.27E-08 |
| Harvest date | SNP_IGA_144919 | Pp02 | 1119192 | 2.27E-08 |
| Harvest date | SNP_IGA_144961 | Pp02 | 1122279 | 2.139E-07 |
| Harvest date | SNP_IGA_145447 | Pp02 | 1149743 | 3.36E-08 |
| Harvest date | SNP_IGA_145505 | Pp02 | 1157565 | 2.27E-08 |
| Harvest date | SNP_IGA_145514 | Pp02 | 1161102 | 2.139E-07 |
| Harvest date | SNP_IGA_145601 | Pp02 | 1166250 | 2.139E-07 |
| Harvest date | SNP_IGA_145717 | Pp02 | 1174968 | 3.36E-08 |
| Harvest date | SNP_IGA_146220 | Pp02 | 1190084 | 2.139E-07 |
| Harvest date | SNP_IGA_146377 | Pp02 | 1200921 | 3.36E-08 |
| Harvest date | SNP_IGA_146426 | Pp02 | 1202511 | 1.15E-08 |
| Harvest date | SNP_IGA_146706 | Pp02 | 1221424 | 2.27E-08 |
| Harvest date | SNP_IGA_146765 | Pp02 | 1223419 | 4.605E-07 |
| Harvest date | SNP_IGA_147219 | Pp02 | 1254917 | 5.37E-08 |
| Harvest date | SNP_IGA_147233 | Pp02 | 1255423 | 5.37E-08 |
| Harvest date | SNP_IGA_147360 | Pp02 | 1276270 | 0.000000004 |
| Harvest date | SNP_IGA_147371 | Pp02 | 1276757 | 3E-10 |
| Harvest date | SNP_IGA_147378 | Pp02 | 1277029 | 3E-10 |
| Harvest date | SNP_IGA_147687 | Pp02 | 1291153 | 3.45E-08 |
| Harvest date | SNP_IGA_147716 | Pp02 | 1292259 | 0.000000004 |
| Harvest date | SNP_IGA_147782 | Pp02 | 1295620 | 3.45E-08 |
| Harvest date | SNP_IGA_148471 | Pp02 | 1346254 | 3E-10 |
| Harvest date | SNP_IGA_148492 | Pp02 | 1346931 | 0.000000004 |
| Harvest date | SNP_IGA_148528 | Pp02 | 1348523 | 6.01E-08 |
| Harvest date | SNP_IGA_148537 | Pp02 | 1348663 | 4.3E-09 |
| Harvest date | SNP_IGA_148598 | Pp02 | 1351253 | 7.4E-09 |
| Harvest date | SNP_IGA_148760 | Pp02 | 1363655 | 2.96E-08 |
| Harvest date | SNP_IGA_148770 | Pp02 | 1364391 | 7.74E-08 |
| Harvest date | SNP_IGA_148777 | Pp02 | 1364858 | 2.96E-08 |
| Harvest date | SNP_IGA_149868 | Pp02 | 1459484 | 7.74E-08 |
| Harvest date | SNP_IGA_150539 | Pp02 | 1519059 | 1.3E-09 |
| Harvest date | SNP_IGA_150673 | Pp02 | 1531581 | 9.12E-08 |
| Harvest date | SNP_IGA_150678 | Pp02 | 1531768 | 7.7E-09 |
| Harvest date | SNP_IGA_150857 | Pp02 | 1552639 | 7.74E-08 |
| Harvest date | SNP_IGA_150874 | Pp02 | 1553038 | 7.74E-08 |
| Harvest date | SNP_IGA_151067 | Pp02 | 1564832 | 3.349E-07 |
| Harvest date | SNP_IGA_151613 | Pp02 | 1643232 | 3.349E-07 |
| Harvest date | SNP_IGA_152082 | Pp02 | 1699475 | 9.262E-07 |
| Harvest date | SNP_IGA_152111 | Pp02 | 1702004 | 3.349E-07 |
| Harvest date | SNP_IGA_152301 | Pp02 | 1715209 | 3.349E-07 |
| Harvest date | SNP_IGA_152320 | Pp02 | 1716750 | 3.236E-07 |
| Harvest date | SNP_IGA_152439 | Pp02 | 1725935 | 1.7E-09 |
| Harvest date | SNP_IGA_152976 | Pp02 | 1761256 | 1.5E-09 |
| Harvest date | SNP_IGA_153388 | Pp02 | 1781488 | 1.5E-09 |
| Harvest date | SNP_IGA_153443 | Pp02 | 1784370 | 1E-10 |
| Harvest date | SNP_IGA_153556 | Pp02 | 1796649 | 3.2E-09 |
| Harvest date | SNP_IGA_153672 | Pp02 | 1802827 | 4.393E-07 |
| Harvest date | SNP_IGA_153686 | Pp02 | 1804532 | 3.2E-09 |
| Harvest date | SNP_IGA_153785 | Pp02 | 1814837 | 4.393E-07 |
| Harvest date | SNP_IGA_154038 | Pp02 | 1827831 | 4.393E-07 |
| Harvest date | SNP_IGA_154259 | Pp02 | 1850663 | 2.4E-09 |
| Harvest date | SNP_IGA_154354 | Pp02 | 1887418 | 3.2E-09 |
| Harvest date | SNP_IGA_154368 | Pp02 | 1887989 | 2.8E-09 |
| Harvest date | SNP_IGA_154383 | Pp02 | 1888630 | 5E-10 |
| Harvest date | SNP_IGA_154391 | Pp02 | 1888715 | 3E-10 |
| Harvest date | SNP_IGA_155412 | Pp02 | 1929182 | 3.7E-09 |
| Harvest date | SNP_IGA_155433 | Pp02 | 1929742 | 3.7E-09 |
| Harvest date | SNP_IGA_155673 | Pp02 | 1947776 | 1.5E-09 |
| Harvest date | SNP_IGA_155680 | Pp02 | 1949477 | 3.7E-09 |
| Harvest date | SNP_IGA_156002 | Pp02 | 1972009 | 3.2E-09 |
| Harvest date | SNP_IGA_156313 | Pp02 | 2008443 | 4E-10 |
| Harvest date | SNP_IGA_156742 | Pp02 | 2037199 | 1.21E-08 |
| Harvest date | SNP_IGA_156774 | Pp02 | 2043178 | 3.2E-09 |
| Harvest date | SNP_IGA_157433 | Pp02 | 2094415 | 1E-10 |
| Harvest date | SNP_IGA_157529 | Pp02 | 2114745 | 2.1898E-06 |
| Harvest date | SNP_IGA_157556 | Pp02 | 2119637 | 1.5E-09 |
| Harvest date | SNP_IGA_157644 | Pp02 | 2134115 | 3.7E-09 |
| Harvest date | SNP_IGA_157737 | Pp02 | 2145946 | 1.5E-09 |
| Harvest date | SNP_IGA_157889 | Pp02 | 2167137 | 6.38E-08 |
| Harvest date | SNP_IGA_158011 | Pp02 | 2186582 | 5.17E-08 |
| Harvest date | SNP_IGA_158017 | Pp02 | 2186975 | 6.38E-08 |
| Harvest date | SNP_IGA_158157 | Pp02 | 2204335 | 2.4599E-06 |
| Harvest date | SNP_IGA_158810 | Pp02 | 2237722 | 0.000000466 |
| Harvest date | SNP_IGA_158884 | Pp02 | 2239970 | 2.26E-08 |
| Harvest date | SNP_IGA_158919 | Pp02 | 2241727 | 0.000000466 |
| Harvest date | SNP_IGA_159649 | Pp02 | 2307132 | 3.725E-07 |
| Harvest date | SNP_IGA_159881 | Pp02 | 2326322 | 2.2712E-06 |
| Harvest date | SNP_IGA_161939 | Pp02 | 2478864 | 1.2589E-06 |
| Harvest date | SNP_IGA_163292 | Pp02 | 2570445 | 1.0416E-06 |
| Harvest date | SNP_IGA_163588 | Pp02 | 2601927 | 1.0416E-06 |
| Harvest date | SNP_IGA_163716 | Pp02 | 2607969 | 1.724E-07 |
| Harvest date | SNP_IGA_163725 | Pp02 | 2608462 | 2.265E-07 |
| Harvest date | SNP_IGA_164374 | Pp02 | 2673561 | 4.83E-08 |
| Harvest date | SNP_IGA_164863 | Pp02 | 2722646 | 6.335E-07 |
| Harvest date | SNP_IGA_164886 | Pp02 | 2727676 | 4.773E-07 |
| Harvest date | SNP_IGA_171044 | Pp02 | 3134298 | 7.101E-07 |
| Harvest date | SNP_IGA_171092 | Pp02 | 3136025 | 7.101E-07 |
| Harvest date | SNP_IGA_171112 | Pp02 | 3137552 | 7.101E-07 |
| Harvest date | SNP_IGA_171141 | Pp02 | 3139384 | 7.101E-07 |
| Harvest date | SNP_IGA_173418 | Pp02 | 3241279 | 7.361E-07 |
| Harvest date | SNP_IGA_174917 | Pp02 | 3310222 | 7.361E-07 |
| Harvest date | SNP_IGA_228997 | Pp02 | 9066783 | 9.7E-09 |
| Harvest date | SNP_IGA_234881 | Pp02 | 9868018 | 0.000000004 |
| Harvest date | SNP_IGA_236039 | Pp02 | 10125154 | 7.12E-08 |
| Harvest date | SNP_IGA_236207 | Pp02 | 10134167 | 3.44E-08 |
| Harvest date | SNP_IGA_236409 | Pp02 | 10144711 | 7.12E-08 |
| Harvest date | SNP_IGA_236414 | Pp02 | 10145001 | 7.12E-08 |
| Harvest date | SNP_IGA_236666 | Pp02 | 10160089 | 7.12E-08 |
| Harvest date | SNP_IGA_287687 | Pp02 | 25227906 | 4.773E-07 |
| Harvest date | SNP_IGA_287700 | Pp02 | 25228844 | 6.37E-08 |
| Anthocyanins | SNP_IGA_181444 | Pp02 | 3800271 | 2E-10 |
| Sorbitol | SNP_IGA_152976 | Pp02 | 1761256 | 6.341E-07 |
| Sorbitol | SNP_IGA_153388 | Pp02 | 1781488 | 6.341E-07 |
| Sorbitol | SNP_IGA_153443 | Pp02 | 1784370 | 6.341E-07 |
| Sorbitol | SNP_IGA_153556 | Pp02 | 1796649 | 1.038E-07 |
| Sorbitol | SNP_IGA_153686 | Pp02 | 1804532 | 1.038E-07 |
| Sorbitol | SNP_IGA_154354 | Pp02 | 1887418 | 1.038E-07 |
| Sorbitol | SNP_IGA_154391 | Pp02 | 1888715 | 8.157E-07 |
| Sorbitol | SNP_IGA_155673 | Pp02 | 1947776 | 6.341E-07 |
| Sorbitol | SNP_IGA_156002 | Pp02 | 1972009 | 1.038E-07 |
| Sorbitol | SNP_IGA_156313 | Pp02 | 2008443 | 1.038E-07 |
| Sorbitol | SNP_IGA_156774 | Pp02 | 2043178 | 1.038E-07 |
| Sorbitol | SNP_IGA_157433 | Pp02 | 2094415 | 6.341E-07 |
| Sorbitol | SNP_IGA_157529 | Pp02 | 2114745 | 2.0355E-06 |
| Sorbitol | SNP_IGA_157556 | Pp02 | 2119637 | 6.341E-07 |
| Sorbitol | SNP_IGA_157737 | Pp02 | 2145946 | 6.341E-07 |
| Sorbitol | SNP_IGA_158884 | Pp02 | 2239970 | 1.5331E-06 |
| Sorbitol | SNP_IGA_164886 | Pp02 | 2727676 | 2.3958E-06 |
| Sorbitol | SNP_IGA_287687 | Pp02 | 25227906 | 2.3958E-06 |
| Sorbitol | SNP_IGA_287700 | Pp02 | 25228844 | 2.3958E-06 |
| Blooming date | SNP_IGA_365780 | Pp03 | 20635992 | 9.567E-07 |
| Harvest date | SNP_IGA_303724 | Pp03 | 4002228 | 2.418E-07 |
| Harvest date | SNP_IGA_309349 | Pp03 | 5659770 | 2.0747E-06 |
| Harvest date | SNP_IGA_363719 | Pp03 | 19759990 | 1.7344E-06 |
| Relative Antioxidant Capacity | SNP_IGA_303724 | Pp03 | 4002228 | 7.245E-07 |
| Blooming date | SNP_IGA_430583 | Pp04 | 15574015 | 1.0476E-06 |
| Blooming date | SNP_IGA_431437 | Pp04 | 15743804 | 1.16E-08 |
| Blooming date | SNP_IGA_441507 | Pp04 | 18422944 | 1.2142E-06 |
| Blooming date | SNP_IGA_441749 | Pp04 | 18496230 | 5.332E-07 |
| Blooming date | SNP_IGA_441887 | Pp04 | 18520777 | 1.2142E-06 |
| Blooming date | SNP_IGA_441904 | Pp04 | 18522596 | 1.2142E-06 |
| Harvest date | SNP_IGA_403353 | Pp04 | 8996802 | 8.379E-07 |
| Harvest date | SNP_IGA_403613 | Pp04 | 9041129 | 0.000000385 |
| Harvest date | SNP_IGA_442063 | Pp04 | 18548028 | 2.5E-09 |
| Harvest date | SNP_IGA_442235 | Pp04 | 18585658 | 2.5E-09 |
| Harvest date | SNP_IGA_442267 | Pp04 | 18590365 | 2.5E-09 |
| Harvest date | SNP_IGA_442526 | Pp04 | 18655371 | 3.84E-08 |
| Harvest date | SNP_IGA_443304 | Pp04 | 18742047 | 2.54E-08 |
| Harvest date | SNP_IGA_443441 | Pp04 | 18770127 | 2.878E-07 |
| Harvest date | SNP_IGA_444204 | Pp04 | 18861832 | 2.54E-08 |
| Harvest date | SNP_IGA_444291 | Pp04 | 18881545 | 2.54E-08 |
| Harvest date | SNP_IGA_446390 | Pp04 | 19397033 | 1.9947E-06 |
| Harvest date | SNP_IGA_448998 | Pp04 | 19896554 | 8E-10 |
| Harvest date | SNP_IGA_449007 | Pp04 | 19897176 | 8E-10 |
| Harvest date | SNP_IGA_449112 | Pp04 | 19905501 | 0 |
| Harvest date | SNP_IGA_450711 | Pp04 | 20165259 | 8E-10 |
| Anthocyanins | SNP_IGA_392956 | Pp04 | 5689470 | 2E-10 |
| Anthocyanins | SNP_IGA_393060 | Pp04 | 5694032 | 2E-10 |
| Anthocyanins | SNP_IGA_393507 | Pp04 | 5726360 | 2E-10 |
| Anthocyanins | SNP_IGA_395202 | Pp04 | 6168570 | 2E-10 |
| Sorbitol | SNP_IGA_442063 | Pp04 | 18548028 | 9.66E-08 |
| Sorbitol | SNP_IGA_442235 | Pp04 | 18585658 | 9.66E-08 |
| Sorbitol | SNP_IGA_442267 | Pp04 | 18590365 | 9.66E-08 |
| Sorbitol | SNP_IGA_443304 | Pp04 | 18742047 | 0.000000146 |
| Sorbitol | SNP_IGA_444204 | Pp04 | 18861832 | 0.000000146 |
| Sorbitol | SNP_IGA_444291 | Pp04 | 18881545 | 0.000000146 |
| Sorbitol | SNP_IGA_448998 | Pp04 | 19896554 | 3.412E-07 |
| Sorbitol | SNP_IGA_449007 | Pp04 | 19897176 | 3.412E-07 |
| Sorbitol | SNP_IGA_449112 | Pp04 | 19905501 | 1.26E-08 |
| Sorbitol | SNP_IGA_450711 | Pp04 | 20165259 | 3.412E-07 |
| Total sugars | SNP_IGA_442063 | Pp04 | 18548028 | 9.786E-07 |
| Total sugars | SNP_IGA_442235 | Pp04 | 18585658 | 9.786E-07 |
| Total sugars | SNP_IGA_442267 | Pp04 | 18590365 | 9.786E-07 |
| Total sugars | SNP_IGA_443304 | Pp04 | 18742047 | 1.7609E-06 |
| Total sugars | SNP_IGA_444204 | Pp04 | 18861832 | 1.7609E-06 |
| Total sugars | SNP_IGA_444291 | Pp04 | 18881545 | 1.7609E-06 |
| Total sugars | SNP_IGA_449112 | Pp04 | 19905501 | 7.096E-07 |
| Harvest date | SNP_IGA_543247 | Pp05 | 276220 | 5.675E-07 |
| Harvest date | SNP_IGA_543786 | Pp05 | 467067 | 7.578E-07 |
| Harvest date | SNP_IGA_543942 | Pp05 | 481014 | 7.578E-07 |
| Harvest date | SNP_IGA_544157 | Pp05 | 521864 | 7.578E-07 |
| Harvest date | SNP_IGA_566064 | Pp05 | 4919872 | 1.7344E-06 |
| Harvest date | SNP_IGA_566086 | Pp05 | 4921618 | 1.7344E-06 |
| Harvest date | SNP_IGA_566097 | Pp05 | 4922376 | 1.7344E-06 |
| Harvest date | SNP_IGA_567030 | Pp05 | 4971864 | 1.7344E-06 |
| Harvest date | SNP_IGA_567045 | Pp05 | 4972718 | 1.7344E-06 |
| Harvest date | SNP_IGA_569241 | Pp05 | 5175645 | 1.7344E-06 |
| Harvest date | SNP_IGA_569485 | Pp05 | 5202012 | 1.7344E-06 |
| Harvest date | SNP_IGA_569773 | Pp05 | 5232302 | 1.7344E-06 |
| Harvest date | SNP_IGA_600691 | Pp05 | 14995466 | 4.97E-08 |
| Harvest date | SNP_IGA_619807 | Pp06 | 4759496 | 0.000000004 |
| Harvest date | SNP_IGA_630302 | Pp06 | 8238299 | 1.9075E-06 |
| Harvest date | SNP_IGA_636280 | Pp06 | 10477001 | 2.0777E-06 |
| Harvest date | SNP_IGA_700469 | Pp06 | 28045174 | 5.271E-07 |
| Flavonoids | SNP_IGA_628833 | Pp06 | 7901344 | 2.13E-08 |
| Flavonoids | SNP_IGA_629027 | Pp06 | 7914628 | 2.13E-08 |
| Flavonoids | SNP_IGA_629062 | Pp06 | 7918349 | 2.13E-08 |
| Flavonoids | SNP_IGA_629558 | Pp06 | 8000787 | 2.21E-08 |
| Flavonoids | SNP_IGA_630243 | Pp06 | 8227954 | 7.84E-08 |
| Flavonoids | SNP_IGA_630266 | Pp06 | 8232664 | 3.114E-07 |
| Flavonoids | SNP_IGA_630302 | Pp06 | 8238299 | 2.706E-07 |
| Flavonoids | SNP_IGA_630550 | Pp06 | 8337946 | 7.35E-08 |
| Flavonoids | SNP_IGA_630662 | Pp06 | 8369151 | 7.35E-08 |
| Flavonoids | SNP_IGA_636280 | Pp06 | 10477001 | 1.8901E-06 |
| Flavonoids | SNP_IGA_637857 | Pp06 | 10733784 | 1.7553E-06 |
| Flavonoids | SNP_IGA_637861 | Pp06 | 10734009 | 1.7553E-06 |
| Flavonoids | SNP_IGA_638036 | Pp06 | 10823366 | 1.7814E-06 |
| Flavonoids | SNP_IGA_638783 | Pp06 | 11004159 | 1.7814E-06 |
| Flavonoids | SNP_IGA_638859 | Pp06 | 11016846 | 1.7814E-06 |
| Sorbitol | SNP_IGA_700469 | Pp06 | 28045174 | 9.341E-07 |
| Total sugars | SNP_IGA_636024 | Pp06 | 10460202 | 2.198E-07 |
| Total sugars | SNP_IGA_636280 | Pp06 | 10477001 | 3.65E-08 |
| Total sugars | SNP_IGA_636292 | Pp06 | 10477315 | 2.198E-07 |
| Total sugars | SNP_IGA_637345 | Pp06 | 10604025 | 2.198E-07 |
| Total sugars | SNP_IGA_637355 | Pp06 | 10606410 | 2.198E-07 |
| Harvest date | SNP_IGA_746619 | Pp07 | 7470226 | 0.000001507 |
| Harvest date | SNP_IGA_746629 | Pp07 | 7471270 | 0.000001507 |
| Harvest date | SNP_IGA_748434 | Pp07 | 7699845 | 1.297E-07 |
| Harvest date | SNP_IGA_749366 | Pp07 | 7830289 | 1.297E-07 |
| Harvest date | SNP_IGA_776214 | Pp07 | 14886166 | 1.375E-07 |
| Harvest date | SNP_IGA_776348 | Pp07 | 14946644 | 1.927E-07 |
| Harvest date | SNP_IGA_779276 | Pp07 | 16145401 | 1.0686E-06 |
| Harvest date | SNP_IGA_783950 | Pp07 | 18388591 | 9.24E-08 |
| Harvest date | SNP_IGA_792898 | Pp07 | 22673209 | 5E-10 |
| Rippening index | SNP_IGA_784373 | Pp07 | 18510773 | 9.434E-07 |
| Rippening index | SNP_IGA_784792 | Pp07 | 18648990 | 8.821E-07 |
| Rippening index | SNP_IGA_784825 | Pp07 | 18680865 | 8.821E-07 |
| Rippening index | SNP_IGA_785228 | Pp07 | 18757412 | 9.434E-07 |
| Rippening index | SNP_IGA_785447 | Pp07 | 18842085 | 8.821E-07 |
| Rippening index | SNP_IGA_786333 | Pp07 | 19334370 | 1.7596E-06 |
| Rippening index | SNP_IGA_786464 | Pp07 | 19405833 | 1.7596E-06 |
| Rippening index | SNP_IGA_786805 | Pp07 | 19508227 | 1.7596E-06 |
| Rippening index | SNP_IGA_786882 | Pp07 | 19522278 | 1.7596E-06 |
| Rippening index | SNP_IGA_786935 | Pp07 | 19542449 | 1.7596E-06 |
| Harvest date | SNP_IGA_797680 | Pp08 | 1271540 | 2.0747E-06 |
| Harvest date | SNP_IGA_806528 | Pp08 | 2986511 | 9E-10 |
| Harvest date | SNP_IGA_806534 | Pp08 | 2986700 | 0.000000004 |
| Harvest date | SNP_IGA_806539 | Pp08 | 2986891 | 9E-10 |
| Harvest date | SNP_IGA_806544 | Pp08 | 2987023 | 0.000000004 |
| Harvest date | SNP_IGA_806557 | Pp08 | 2987480 | 0.000000004 |
| Harvest date | SNP_IGA_806575 | Pp08 | 2987760 | 0.000000004 |
| Harvest date | SNP_IGA_806585 | Pp08 | 2988121 | 9E-10 |
| Harvest date | SNP_IGA_806590 | Pp08 | 2988272 | 0.000000004 |
| Harvest date | SNP_IGA_864149 | Pp08 | 13756987 | 1.1382E-06 |
| Harvest date | SNP_IGA_878210 | Pp08 | 17931190 | 0.000000042 |
| Harvest date | SNP_IGA_878717 | Pp08 | 18085149 | 2E-10 |
| Harvest date | SNP_IGA_878831 | Pp08 | 18117446 | 1.9385E-06 |
| Harvest date | SNP_IGA_878981 | Pp08 | 18179927 | 9E-10 |
| Harvest date | SNP_IGA_879061 | Pp08 | 18219533 | 9E-10 |
| Harvest date | SNP_IGA_879131 | Pp08 | 18245683 | 9E-10 |
| Harvest date | SNP_IGA_879224 | Pp08 | 18309578 | 7.02E-08 |
| Sorbitol | SNP_IGA_878717 | Pp08 | 18085149 | 2.97E-08 |
| Sorbitol | SNP_IGA_878981 | Pp08 | 18179927 | 2.387E-07 |
| Sorbitol | SNP_IGA_879061 | Pp08 | 18219533 | 2.387E-07 |
| Sorbitol | SNP_IGA_879131 | Pp08 | 18245683 | 2.387E-07 |
| Sorbitol | SNP_IGA_879224 | Pp08 | 18309578 | 7.114E-07 |
| Total sugars | SNP_IGA_870629 | Pp08 | 15787171 | 2.3052E-06 |
| Total sugars | SNP_IGA_879224 | Pp08 | 18309578 | 1.5839E-06 |
