# Supplementary material for: Association Mapping Analysis for Fruit Quality Traits in Prunus persica Using SNP Markers
Source: Front Plant Sci. 2019 Jan 17;9:2005. doi: 10.3389/fpls.2018.02005 (PMC6344403; doi:10.3389/fpls.2018.02005)
Supplement: Supplementary file 2 [file Table_2.DOCX]

Supplementary file 2. Gene annotation of the most significant single nucleotide polymorphisms (SNPs) associated with fruit quality in peach

| **Scaffold** | **Position in peach**  **v1.0 (bp)** | **Trait associated** | **Gene ID** | **Arabidopsis**  **genes** | **NCBI Homology** |
| --- | --- | --- | --- | --- | --- |
| Pp01 | [12641008..12642923](http://www.rosaceae.org/gb/gbrowse/prunus_persica?name=scaffold_1:12641008..12642923&enable=NCBI%20Sequence%20Alignments) | BD | ppa006172m | AT5G61930.1 | Arabidopsis thaliana protein of unknown function (DUF794) |
| Pp01 | [14975776..14987708](http://www.rosaceae.org/gb/gbrowse/prunus_persica?name=scaffold_1:14975776..14987708&enable=NCBI%20Sequence%20Alignments) | HD | ppa001794m | AT1G05790.1 | Lipase class 3 family protein |
| Pp01 | [15844403..15844760](http://www.rosaceae.org/gb/gbrowse/prunus_persica?name=scaffold_1:15844403..15844760&enable=NCBI%20Sequence%20Alignments) | HD, anthoc, RAC | ppa026056m | AT3G23200.1 | Uncharacterised protein family (UPF0497) |
| Pp01 | [15890364..15891805](http://www.rosaceae.org/gb/gbrowse/prunus_persica?name=scaffold_1:15890364..15891805&enable=NCBI%20Sequence%20Alignments) | HD, anthoc, RAC | ppa006119m | AT5G48040.1 | Ubiquitin carboxyl-terminal hydrolase family protein |
| Pp01 | [15903823..15911269](http://www.rosaceae.org/gb/gbrowse/prunus_persica?name=scaffold_1:15903823..15911269&enable=NCBI%20Sequence%20Alignments) | HD, anthoc, RAC | ppa005374m | AT3G59300.1 | Pentatricopeptide repeat (PPR) superfamily protein |
| Pp01 | [16389061..16392182](http://www.rosaceae.org/gb/gbrowse/prunus_persica?name=scaffold_1:16389061..16392182&enable=NCBI%20Sequence%20Alignments) | HD, anthoc, RAC | ppa022257m | AT1G16120.1 | wall associated kinase-like 1 |
| Pp01 | [18571159..18577109](http://www.rosaceae.org/gb/gbrowse/prunus_persica?name=scaffold_1:18571159..18577109&enable=NCBI%20Sequence%20Alignments) | HD, anthoc, RAC | ppa000933m | AT2G43500.1 | Plant regulator RWP-RK family protein |
| Pp01 | [18601904..18604363](http://www.rosaceae.org/gb/gbrowse/prunus_persica?name=scaffold_1:18601904..18604363&enable=NCBI%20Sequence%20Alignments) | HD, anthoc, RAC | ppa002097m | AT3G54540.1 | General control non-repressible 4 |
| Pp01 | [18621268..18624310](http://www.rosaceae.org/gb/gbrowse/prunus_persica?name=scaffold_1:18621268..18624310&enable=NCBI%20Sequence%20Alignments) | HD, anthoc, RAC | ppa020377m | AT3G47570.1 | Leucine-rich repeat protein kinase family protein |
| Pp01 | [18760246..18763009](http://www.rosaceae.org/gb/gbrowse/prunus_persica?name=scaffold_1:18760246..18763009&enable=NCBI%20Sequence%20Alignments) | HD, anthoc, RAC | ppa023496m | AT5G20480.1 | EF-TU receptor |
| Pp01 | [18808165..18811367](http://www.rosaceae.org/gb/gbrowse/prunus_persica?name=scaffold_1:18808165..18811367&enable=NCBI%20Sequence%20Alignments) | HD, anthoc, RAC | ppa017947m | AT3G47570.1 | Leucine-rich repeat protein kinase family protein |
| Pp01 | [18910539..18913804](http://www.rosaceae.org/gb/gbrowse/prunus_persica?name=scaffold_1:18910539..18913804&enable=NCBI%20Sequence%20Alignments) | HD, anthoc, RAC | ppb015931m | AT4G05360.1 | Zinc knuckle (CCHC-type) family protein |
| Pp01 | [19082507..19086932](http://www.rosaceae.org/gb/gbrowse/prunus_persica?name=scaffold_1:19082507..19086932&enable=NCBI%20Sequence%20Alignments) | HD, anthoc, RAC | ppa016864m | AT1G65810.1 | P-loop containing nucleoside triphosphate superfamily protein |
| Pp01 | [23721367..23723279](http://www.rosaceae.org/gb/gbrowse/prunus_persica?name=scaffold_1:23721367..23723279&enable=NCBI%20Sequence%20Alignments) | HD, anthoc, RAC | ppa016499m | AT1G27900.1 | RNA helicase family protein |
| Pp01 | [25342080..25345325](http://www.rosaceae.org/gb/gbrowse/prunus_persica?name=scaffold_1:25342080..25345325&enable=NCBI%20Sequence%20Alignments) | HD, anthoc, Flavonoids, RAC | ppa005996m | AT4G32180.3 | Pantothenate kinase 2 |
| Pp01 | [27648842..27652287](http://www.rosaceae.org/gb/gbrowse/prunus_persica?name=scaffold_1:27648842..27652287&enable=NCBI%20Sequence%20Alignments) | HD, anthoc, Flavonoids, RAC | ppa002650m | AT1G13860.3 | QUASIMODO2 LIKE 1 |
| Pp01 | [27676963..27678258](http://www.rosaceae.org/gb/gbrowse/prunus_persica?name=scaffold_1:27676963..27678258&enable=NCBI%20Sequence%20Alignments) | HD, anthoc, Flavonoids, RAC | ppa019451m | AT5G62660.1 | F-box and associated interaction domains-containing protein |
| Pp01 | [27714258..27716847](http://www.rosaceae.org/gb/gbrowse/prunus_persica?name=scaffold_1:27714258..27716847&enable=NCBI%20Sequence%20Alignments) | HD, anthoc, Flavonoids, RAC | ppa014966m | AT1G13820.1 | alpha/beta-Hydrolases superfamily protein |
| Pp01 | [27814887..27815252](http://www.rosaceae.org/gb/gbrowse/prunus_persica?name=scaffold_1:27814887..27815252&enable=NCBI%20Sequence%20Alignments) | HD, anthoc, Flavonoids, RAC | ppa023911m | AT1G17130.1 | Family of unknown function (DUF572) |
| Pp01 | [27821285..27824767](http://www.rosaceae.org/gb/gbrowse/prunus_persica?name=scaffold_1:27821285..27824767&enable=NCBI%20Sequence%20Alignments) | HD, anthoc, Flavonoids, RAC | ppa026942m | AT2G03670.1 | Cell division cycle 48B |
| Pp01 | [28532994..28534266](http://www.rosaceae.org/gb/gbrowse/prunus_persica?name=scaffold_1:28532994..28534266&enable=NCBI%20Sequence%20Alignments) | HD, anthoc, Flavonoids, RAC | ppa024890m | AT1G13520.1 | Protein of unknown function (DUF1262) |
| Pp01 | [28546498..28547551](http://www.rosaceae.org/gb/gbrowse/prunus_persica?name=scaffold_1:28546498..28547551&enable=NCBI%20Sequence%20Alignments) | HD, anthoc, Flavonoids, RAC | ppa019157m | AT1G53550.1 | F-box family protein |
| Pp01 | [28549551..28552893](http://www.rosaceae.org/gb/gbrowse/prunus_persica?name=scaffold_1:28549551..28552893&enable=NCBI%20Sequence%20Alignments) | HD, anthoc, Flavonoids, RAC | ppa020120m | AT4G29090.1 | Ribonuclease H-like superfamily protein |
| Pp01 | [28661391..28666431](http://www.rosaceae.org/gb/gbrowse/prunus_persica?name=scaffold_1:28661391..28666431&enable=NCBI%20Sequence%20Alignments) | HD, Flavonoids, RAC | ppa000420m | AT1G68710.1 | ATPase E1-E2 type family protein |
| Pp01 | [28673586..28678668](http://www.rosaceae.org/gb/gbrowse/prunus_persica?name=scaffold_1:28673586..28678668&enable=NCBI%20Sequence%20Alignments) | HD, Flavonoids, RAC | ppa000186m | AT1G68720.1 | tRNA arginine adenosine deaminase |
| Pp01 | [35576892..35578233](http://www.rosaceae.org/gb/gbrowse/prunus_persica?name=scaffold_1:35576892..35578233&enable=NCBI%20Sequence%20Alignments) | HD, RAC | ppa022224m | AT2G14760.1 | Basic helix-loop-helix (bHLH) DNA-binding superfamily protein |
| Pp01 | [35614141..35618798](http://www.rosaceae.org/gb/gbrowse/prunus_persica?name=scaffold_1:35614141..35618798&enable=NCBI%20Sequence%20Alignments) | HD, Flavonoids, RAC | ppa002663m | AT1G49890.1 | Family of unknown function (DUF566) |
| Pp01 | [35797353..35801120](http://www.rosaceae.org/gb/gbrowse/prunus_persica?name=scaffold_1:35797353..35801120&enable=NCBI%20Sequence%20Alignments) | HD, Flavonoids, RAC | ppa000860m | AT3G47570.1 | Leucine-rich repeat protein kinase family protein |
| Pp01 | [36067677..36069915](http://www.rosaceae.org/gb/gbrowse/prunus_persica?name=scaffold_1:36067677..36069915&enable=NCBI%20Sequence%20Alignments) | HD, Flavonoids, RAC | ppa021918m | AT4G36930.1 | Basic helix-loop-helix (bHLH) DNA-binding superfamily protein |
| Pp01 | [36096174..36098477](http://www.rosaceae.org/gb/gbrowse/prunus_persica?name=scaffold_1:36096174..36098477&enable=NCBI%20Sequence%20Alignments) | HD, Flavonoids, RAC | ppa001770m | AT5G67090.1 | Subtilisin-like serine endopeptidase family protein |
| Pp01 | [36758296..36762609](http://www.rosaceae.org/gb/gbrowse/prunus_persica?name=scaffold_1:36758296..36762609&enable=NCBI%20Sequence%20Alignments) | HD, Flavonoids, RAC | ppa015943m | AT1G33390.1 | RNA helicase family protein |
| Pp01 | [44934218..44937734](http://www.rosaceae.org/gb/gbrowse/prunus_persica?name=scaffold_1:44934218..44937734&enable=NCBI%20Sequence%20Alignments) | HD | ppa007498m | AT1G76690.1 | 12-oxophytodienoate reductase 2 |
| Pp02 | [1009896..1012195](http://www.rosaceae.org/gb/gbrowse/prunus_persica?name=scaffold_2:1009896..1012195&enable=NCBI%20Sequence%20Alignments) | HD | ppa004120m | AT3G63440.1 | cytokinin oxidase/dehydrogenase 6 |
| Pp02 | [10125057..10128957](http://www.rosaceae.org/gb/gbrowse/prunus_persica?name=scaffold_2:10125057..10128957&enable=NCBI%20Sequence%20Alignments) | HD, Sorbitol | ppa006367m | AT1G04945.3 | HIT-type Zinc finger family protein |
| Pp02 | [10142019..10146169](http://www.rosaceae.org/gb/gbrowse/prunus_persica?name=scaffold_2:10142019..10146169&enable=NCBI%20Sequence%20Alignments) | HD, Sorbitol | ppa002321m | AT2G32850.2 | Protein kinase superfamily protein |
| Pp02 | [10158916..10161595](http://www.rosaceae.org/gb/gbrowse/prunus_persica?name=scaffold_2:10158916..10161595&enable=NCBI%20Sequence%20Alignments) | HD, Sorbitol | ppa016605m | AT5G67030.1 | Zeaxanthin epoxidase (ZEP) (ABA1) |
| Pp02 | [1021940..1024036](http://www.rosaceae.org/gb/gbrowse/prunus_persica?name=scaffold_2:1021940..1024036&enable=NCBI%20Sequence%20Alignments) | HD | ppa021176m | AT5G13460.1 | IQ-domain 11 |
| Pp02 | [1033285..1035450](http://www.rosaceae.org/gb/gbrowse/prunus_persica?name=scaffold_2:1033285..1035450&enable=NCBI%20Sequence%20Alignments) | HD | ppa023145m | AT4G20740.1 | Pentatricopeptide repeat (PPR-like) superfamily protein |
| Pp02 | [1052835..1058100](http://www.rosaceae.org/gb/gbrowse/prunus_persica?name=scaffold_2:1052835..1058100&enable=NCBI%20Sequence%20Alignments) | HD | ppa016901m | AT4G27190.1 | NB-ARC domain-containing disease resistance protein |
| Pp02 | [1116245..1119384](http://www.rosaceae.org/gb/gbrowse/prunus_persica?name=scaffold_2:1116245..1119384&enable=NCBI%20Sequence%20Alignments) | HD | ppa019511m | AT2G13620.1 | Cation/hydrogen exchanger 15 |
| Pp02 | [1120070..1127447](http://www.rosaceae.org/gb/gbrowse/prunus_persica?name=scaffold_2:1120070..1127447&enable=NCBI%20Sequence%20Alignments) | HD | ppa004008m | AT3G08550.1 | Elongation defective 1 protein / ELD1 protein |
| Pp02 | [1149059..1150339](http://www.rosaceae.org/gb/gbrowse/prunus_persica?name=scaffold_2:1149059..1150339&enable=NCBI%20Sequence%20Alignments) | HD | ppa021557m | AT3G51930.1 | Transducin/WD40 repeat-like superfamily protein |
| Pp02 | [1157298..1161347](http://www.rosaceae.org/gb/gbrowse/prunus_persica?name=scaffold_2:1157298..1161347&enable=NCBI%20Sequence%20Alignments) | HD | ppa024377m | AT3G14460.1 | LRR and NB-ARC domains-containing disease resistance protein |
| Pp02 | [1165914..1166261](http://www.rosaceae.org/gb/gbrowse/prunus_persica?name=scaffold_2:1165914..1166261&enable=NCBI%20Sequence%20Alignments) | HD | ppa023519m | AT4G30880.1 | Bifunctional inhibitor/lipid-transfer protein/seed superfamily protein |
| Pp02 | [1174308..1175205](http://www.rosaceae.org/gb/gbrowse/prunus_persica?name=scaffold_2:1174308..1175205&enable=NCBI%20Sequence%20Alignments) | HD | ppa025702m | AT4G30880.1 | Bifunctional inhibitor/lipid-transfer protein/seed superfamily protein |
| Pp02 | [1188729..1190329](http://www.rosaceae.org/gb/gbrowse/prunus_persica?name=scaffold_2:1188729..1190329&enable=NCBI%20Sequence%20Alignments) | HD | ppa004557m | AT1G05600.2 | Tetratricopeptide repeat (TPR)-like superfamily protein |
| Pp02 | [1200633..1203229](http://www.rosaceae.org/gb/gbrowse/prunus_persica?name=scaffold_2:1200633..1203229&enable=NCBI%20Sequence%20Alignments) | HD | ppa022675m | AT1G64260.1 | MuDR family transposase |
| Pp02 | [1220843..1227605](http://www.rosaceae.org/gb/gbrowse/prunus_persica?name=scaffold_2:1220843..1227605&enable=NCBI%20Sequence%20Alignments) | HD | ppa002700m | AT5G50400.1 | Purple acid phosphatase 27 |
| Pp02 | [1254912..1255463](http://www.rosaceae.org/gb/gbrowse/prunus_persica?name=scaffold_2:1254912..1255463&enable=NCBI%20Sequence%20Alignments) | HD | ppa019297m | AT2G21100.1 | Disease resistance-responsive (dirigent-like protein) family protein |
| Pp02 | [1275505..1277546](http://www.rosaceae.org/gb/gbrowse/prunus_persica?name=scaffold_2:1275505..1277546&enable=NCBI%20Sequence%20Alignments) | HD | ppa020586m | AT3G44900.1 | Cation/H+ exchanger 4 |
| Pp02 | [1290725..1295853](http://www.rosaceae.org/gb/gbrowse/prunus_persica?name=scaffold_2:1290725..1295853&enable=NCBI%20Sequence%20Alignments) | HD | ppa014872m | AT4G26090.1 | NB-ARC domain-containing disease resistance protein |
| Pp02 | [1346116..1352993](http://www.rosaceae.org/gb/gbrowse/prunus_persica?name=scaffold_2:1346116..1352993&enable=NCBI%20Sequence%20Alignments) | HD | ppb017543m | AT4G27190.1 | NB-ARC domain-containing disease resistance protein |
| Pp02 | [1363073..1366219](http://www.rosaceae.org/gb/gbrowse/prunus_persica?name=scaffold_2:1363073..1366219&enable=NCBI%20Sequence%20Alignments) | HD | ppa001109m | AT2G38800.1 | Plant calmodulin-binding protein-related |
| Pp02 | [1454585..1466025](http://www.rosaceae.org/gb/gbrowse/prunus_persica?name=scaffold_2:1454585..1466025&enable=NCBI%20Sequence%20Alignments) | HD | ppa000166m | AT2G38770.1 | P-loop containing nucleoside triphosphate superfamily protein |
| Pp02 | [1517412..1519926](http://www.rosaceae.org/gb/gbrowse/prunus_persica?name=scaffold_2:1517412..1519926&enable=NCBI%20Sequence%20Alignments) | HD | ppb025046m | AT1G64260.1 | MuDR family transposase |
| Pp02 | [1531223..1531931](http://www.rosaceae.org/gb/gbrowse/prunus_persica?name=scaffold_2:1531223..1531931&enable=NCBI%20Sequence%20Alignments) | HD | ppa018116m | AT5G16360.1 | NC domain-containing protein-related |
| Pp02 | [1552271..1553397](http://www.rosaceae.org/gb/gbrowse/prunus_persica?name=scaffold_2:1552271..1553397&enable=NCBI%20Sequence%20Alignments) | HD | ppa016259m | AT4G38180.1 | FAR1-related sequence 5 |
| Pp02 | [1558578..1567548](http://www.rosaceae.org/gb/gbrowse/prunus_persica?name=scaffold_2:1558578..1567548&enable=NCBI%20Sequence%20Alignments) | HD | ppa017744m | AT3G54510.2 | Early-responsive to dehydration stress protein (ERD4) |
| Pp02 | [1639392..1643280](http://www.rosaceae.org/gb/gbrowse/prunus_persica?name=scaffold_2:1639392..1643280&enable=NCBI%20Sequence%20Alignments) | HD | ppa007078m | AT1G18480.1 | Calcineurin-like metallo-phosphoesterase superfamily protein |
| Pp02 | [1698182..1700324](http://www.rosaceae.org/gb/gbrowse/prunus_persica?name=scaffold_2:1698182..1700324&enable=NCBI%20Sequence%20Alignments) | HD | ppa008922m | AT2G38750.1 | Annexin 4 |
| Pp02 | [1701684..1703900](http://www.rosaceae.org/gb/gbrowse/prunus_persica?name=scaffold_2:1701684..1703900&enable=NCBI%20Sequence%20Alignments) | HD | ppa008921m | AT5G12380.1 | Annexin 8 |
| Pp02 | [1701684..1704264](http://www.rosaceae.org/gb/gbrowse/prunus_persica?name=scaffold_2:1701684..1704264&enable=NCBI%20Sequence%20Alignments) | HD | ppa008912m | AT5G12380.1 | Annexin 8 |
| Pp02 | [1724337..1727752](http://www.rosaceae.org/gb/gbrowse/prunus_persica?name=scaffold_2:1724337..1727752&enable=NCBI%20Sequence%20Alignments) | HD | ppa018371m | AT3G54490.1 | RNA polymerase II fifth largest subunit, E |
| Pp02 | [1760807..1761627](http://www.rosaceae.org/gb/gbrowse/prunus_persica?name=scaffold_2:1760807..1761627&enable=NCBI%20Sequence%20Alignments) | HD, Sorbitol | ppa024158m | AT3G14460.1 | LRR and NB-ARC domains-containing disease resistance protein |
| Pp02 | [1781289..1784883](http://www.rosaceae.org/gb/gbrowse/prunus_persica?name=scaffold_2:1781289..1784883&enable=NCBI%20Sequence%20Alignments) | HD, Sorbitol | ppa004992m | AT3G54470.1 | Uridine 5\'-monophosphate synthase / UMP (PYRE-F) (UMPS) |
| Pp02 | [1795218..1797305](http://www.rosaceae.org/gb/gbrowse/prunus_persica?name=scaffold_2:1795218..1797305&enable=NCBI%20Sequence%20Alignments) | HD, Sorbitol | ppa025139m | AT5G59100.1 | Subtilisin-like serine endopeptidase family protein |
| Pp02 | [1802711..1804831](http://www.rosaceae.org/gb/gbrowse/prunus_persica?name=scaffold_2:1802711..1804831&enable=NCBI%20Sequence%20Alignments) | HD, Sorbitol | ppa022813m | AT5G59100.1 | Subtilisin-like serine endopeptidase family protein |
| Pp02 | [1813055..1815160](http://www.rosaceae.org/gb/gbrowse/prunus_persica?name=scaffold_2:1813055..1815160&enable=NCBI%20Sequence%20Alignments) | HD, Sorbitol | ppa024105m | AT5G59100.1 | Subtilisin-like serine endopeptidase family protein |
| Pp02 | [1850616..1851100](http://www.rosaceae.org/gb/gbrowse/prunus_persica?name=scaffold_2:1850616..1851100&enable=NCBI%20Sequence%20Alignments) | HD, Sorbitol | ppa025134m | AT3G22170.1 | Far-red elongated hypocotyls 3 |
| Pp02 | [1887404..1888786](http://www.rosaceae.org/gb/gbrowse/prunus_persica?name=scaffold_2:1887404..1888786&enable=NCBI%20Sequence%20Alignments) | HD, Sorbitol | ppa021336m | AT5G60800.1 | Heavy metal transport/detoxification superfamily protein |
| Pp02 | [1928931..1930729](http://www.rosaceae.org/gb/gbrowse/prunus_persica?name=scaffold_2:1928931..1930729&enable=NCBI%20Sequence%20Alignments) | HD, Sorbitol | ppa022579m | AT4G38180.1 | FAR1-related sequence 5 |
| Pp02 | [1947168..1951535](http://www.rosaceae.org/gb/gbrowse/prunus_persica?name=scaffold_2:1947168..1951535&enable=NCBI%20Sequence%20Alignments) | HD, Sorbitol | ppa021061m | AT3G14460.1 | LRR and NB-ARC domains-containing disease resistance protein |
| Pp02 | [1969894..1975484](http://www.rosaceae.org/gb/gbrowse/prunus_persica?name=scaffold_2:1969894..1975484&enable=NCBI%20Sequence%20Alignments) | HD, Sorbitol | ppa015535m | AT3G54460.1 | SNF2 domain-containing protein |
| Pp02 | [2006926..2009863](http://www.rosaceae.org/gb/gbrowse/prunus_persica?name=scaffold_2:2006926..2009863&enable=NCBI%20Sequence%20Alignments) | HD, Sorbitol | ppa006469m | AT3G54450.1 | Major facilitator superfamily protein |
| Pp02 | [2037192..2039636](http://www.rosaceae.org/gb/gbrowse/prunus_persica?name=scaffold_2:2037192..2039636&enable=NCBI%20Sequence%20Alignments) | HD, Sorbitol | ppa026521m | AT5G59100.1 | Subtilisin-like serine endopeptidase family protein |
| Pp02 | [2040664..2044301](http://www.rosaceae.org/gb/gbrowse/prunus_persica?name=scaffold_2:2040664..2044301&enable=NCBI%20Sequence%20Alignments) | HD, Sorbitol | ppa003298m | AT3G54450.1 | Major facilitator superfamily protein |
| [Pp02](http://www.rosaceae.org/gb/gbrowse/prunus_persica?name=scaffold_2:2089309..2103526&enable=NCBI%20Sequence%20Alignments) | 2089309..2103526 | HD, Sorbitol | ppa000088m | AT2G40030.1 | Nuclear RNA polymerase D1B |
| Pp02 | [2104602..2115599](http://www.rosaceae.org/gb/gbrowse/prunus_persica?name=scaffold_2:2104602..2115599&enable=NCBI%20Sequence%20Alignments) | HD, Sorbitol | ppa000508m | AT3G54440.1 | Glycoside hydrolase family 2 protein |
| Pp02 | [2115851..2123681](http://www.rosaceae.org/gb/gbrowse/prunus_persica?name=scaffold_2:2115851..2123681&enable=NCBI%20Sequence%20Alignments) | HD, Sorbitol | ppa024567m | AT2G40030.1 | Nuclear RNA polymerase D1B |
| Pp02 | [2126201..2137543](http://www.rosaceae.org/gb/gbrowse/prunus_persica?name=scaffold_2:2126201..2137543&enable=NCBI%20Sequence%20Alignments) | HD, Sorbitol | ppa000532m | AT3G54440.1 | Glycoside hydrolase family 2 protein |
| Pp02 | [2144897..2147544](http://www.rosaceae.org/gb/gbrowse/prunus_persica?name=scaffold_2:2144897..2147544&enable=NCBI%20Sequence%20Alignments) | HD, Sorbitol | ppa021068m | AT2G20060.1 | Ribosomal protein L4/L1 family |
| Pp02 | [2164158..2172210](http://www.rosaceae.org/gb/gbrowse/prunus_persica?name=scaffold_2:2164158..2172210&enable=NCBI%20Sequence%20Alignments) | HD, Sorbitol | ppa003990m | AT3G55850.2 | Amidohydrolase family |
| Pp02 | [2186448..2188155](http://www.rosaceae.org/gb/gbrowse/prunus_persica?name=scaffold_2:2186448..2188155&enable=NCBI%20Sequence%20Alignments) | HD | ppa023899m | AT3G14470.1 | NB-ARC domain-containing disease resistance protein |
| Pp02 | [2203834..2205232](http://www.rosaceae.org/gb/gbrowse/prunus_persica?name=scaffold_2:2203834..2205232&enable=NCBI%20Sequence%20Alignments) | HD, Sorbitol | ppa023084m | AT5G07900.1 | Mitochondrial transcription termination factor family protein |
| Pp02 | [2241298..2242699](http://www.rosaceae.org/gb/gbrowse/prunus_persica?name=scaffold_2:2241298..2242699&enable=NCBI%20Sequence%20Alignments) | HD, Sorbitol | ppa006699m | AT5G07900.1 | Mitochondrial transcription termination factor family protein |
| Pp02 | [2322457..2326386](http://www.rosaceae.org/gb/gbrowse/prunus_persica?name=scaffold_2:2322457..2326386&enable=NCBI%20Sequence%20Alignments) | HD, Sorbitol | ppa014576m | AT3G14470.1 | NB-ARC domain-containing disease resistance protein |
| Pp02 | [25227476..25231046](http://www.rosaceae.org/gb/gbrowse/prunus_persica?name=scaffold_2:25227476..25231046&enable=NCBI%20Sequence%20Alignments) | HD, Sorbitol | ppa014682m | AT4G00350.1 | MATE efflux family protein |
| Pp02 | [2568013..2571265](http://www.rosaceae.org/gb/gbrowse/prunus_persica?name=scaffold_2:2568013..2571265&enable=NCBI%20Sequence%20Alignments) | HD, Sorbitol | ppa020763m | AT1G64260.1 | MuDR family transposase |
| Pp02 | [2600854..2602860](http://www.rosaceae.org/gb/gbrowse/prunus_persica?name=scaffold_2:2600854..2602860&enable=NCBI%20Sequence%20Alignments) | HD, Sorbitol | ppa019826m | AT1G60500.1 | Dynamin related protein 4C |
| Pp02 | [2607179..2609175](http://www.rosaceae.org/gb/gbrowse/prunus_persica?name=scaffold_2:2607179..2609175&enable=NCBI%20Sequence%20Alignments) | HD, Sorbitol | ppa018625m | AT1G60500.1 | Dynamin related protein 4C |
| Pp02 | [2673323..2674189](http://www.rosaceae.org/gb/gbrowse/prunus_persica?name=scaffold_2:2673323..2674189&enable=NCBI%20Sequence%20Alignments) | HD, Sorbitol | ppa017869m | AT5G07900.1 | Mitochondrial transcription termination factor family protein |
| Pp02 | [2719723..2723640](http://www.rosaceae.org/gb/gbrowse/prunus_persica?name=scaffold_2:2719723..2723640&enable=NCBI%20Sequence%20Alignments) | HD, Sorbitol | ppa000407m | AT3G14470.1 | NB-ARC domain-containing disease resistance protein |
| Pp02 | [2724955..2737659](http://www.rosaceae.org/gb/gbrowse/prunus_persica?name=scaffold_2:2724955..2737659&enable=NCBI%20Sequence%20Alignments) | HD, Sorbitol | ppa000149m | AT5G13530.1 | Protein kinases;ubiquitin-protein ligases |
| Pp02 | [3309169..3312439](http://www.rosaceae.org/gb/gbrowse/prunus_persica?name=scaffold_2:3309169..3312439&enable=NCBI%20Sequence%20Alignments) | HD, Sorbitol | ppa020421m | AT5G17680.1 | Disease resistance protein (TIR-NBS-LRR class), putative |
| Pp02 | [460813..463136](http://www.rosaceae.org/gb/gbrowse/prunus_persica?name=scaffold_2:460813..463136&enable=NCBI%20Sequence%20Alignments) | HD | ppa024380m | AT1G10000.1 | Ribonuclease H-like superfamily protein |
| Pp02 | [472157..477921](http://www.rosaceae.org/gb/gbrowse/prunus_persica?name=scaffold_2:472157..477921&enable=NCBI%20Sequence%20Alignments) | HD | ppa000274m | AT3G14460.1 | LRR and NB-ARC domains-containing disease resistance protein |
| Pp02 | [512337..513333](http://www.rosaceae.org/gb/gbrowse/prunus_persica?name=scaffold_2:512337..513333&enable=NCBI%20Sequence%20Alignments) | HD | ppa008924m | AT1G01550.1 | Protein of unknown function (DUF793) |
| Pp02 | [529051..533255](http://www.rosaceae.org/gb/gbrowse/prunus_persica?name=scaffold_2:529051..533255&enable=NCBI%20Sequence%20Alignments) | HD | ppa016937m | AT3G14470.1 | NB-ARC domain-containing disease resistance protein |
| Pp02 | [653638..654511](http://www.rosaceae.org/gb/gbrowse/prunus_persica?name=scaffold_2:653638..654511&enable=NCBI%20Sequence%20Alignments) | HD | ppa019263m | AT4G02050.1 | Sugar transporter protein 7 |
| Pp02 | [692008..694764](http://www.rosaceae.org/gb/gbrowse/prunus_persica?name=scaffold_2:692008..694764&enable=NCBI%20Sequence%20Alignments) | HD | ppa020131m | AT2G28420.1 | Lactoylglutathione lyase / glyoxalase I family protein |
| Pp02 | [711597..713848](http://www.rosaceae.org/gb/gbrowse/prunus_persica?name=scaffold_2:711597..713848&enable=NCBI%20Sequence%20Alignments) | HD | ppa004965m | AT3G51990.1 | Protein kinase superfamily protein |
| Pp02 | [732093..733181](http://www.rosaceae.org/gb/gbrowse/prunus_persica?name=scaffold_2:732093..733181&enable=NCBI%20Sequence%20Alignments) | HD | ppa019942m | AT3G51970.1 | acyl-CoA sterol acyl transferase 1 |
| Pp02 | [741112..745478](http://www.rosaceae.org/gb/gbrowse/prunus_persica?name=scaffold_2:741112..745478&enable=NCBI%20Sequence%20Alignments) | HD | ppa004221m | AT5G13800.1 | Pheophytinase |
| Pp02 | [748365..749068](http://www.rosaceae.org/gb/gbrowse/prunus_persica?name=scaffold_2:748365..749068&enable=NCBI%20Sequence%20Alignments) | HD | ppa026469m | AT3G24255.1 | RNA-directed DNA polymerase (reverse transcriptase)-related |
| Pp02 | [750563..755927](http://www.rosaceae.org/gb/gbrowse/prunus_persica?name=scaffold_2:750563..755927&enable=NCBI%20Sequence%20Alignments) | HD | ppa015499m | AT4G27220.1 | NB-ARC domain-containing disease resistance protein |
| Pp02 | [773609..780701](http://www.rosaceae.org/gb/gbrowse/prunus_persica?name=scaffold_2:773609..780701&enable=NCBI%20Sequence%20Alignments) | HD | ppa026846m | AT4G27220.1 | NB-ARC domain-containing disease resistance protein |
| Pp02 | [9066138..9070101](http://www.rosaceae.org/gb/gbrowse/prunus_persica?name=scaffold_2:9066138..9070101&enable=NCBI%20Sequence%20Alignments) | HD, Sorbitol | ppa026750m | AT3G18670.1 | Ankyrin repeat family protein |
| Pp02 | [911422..912156](http://www.rosaceae.org/gb/gbrowse/prunus_persica?name=scaffold_2:911422..912156&enable=NCBI%20Sequence%20Alignments) | HD | ppa019868m | AT4G35040.1 | Basic-leucine zipper (bZIP) transcription factor family protein |
| Pp02 | [939865..942811](http://www.rosaceae.org/gb/gbrowse/prunus_persica?name=scaffold_2:939865..942811&enable=NCBI%20Sequence%20Alignments) | HD | ppa023263m | AT3G51950.1 | Zinc finger (CCCH-type) family protein |
| Pp02 | [955919..962128](http://www.rosaceae.org/gb/gbrowse/prunus_persica?name=scaffold_2:955919..962128&enable=NCBI%20Sequence%20Alignments) | HD | ppa018972m | AT2G41560.1 | Autoinhibited Ca(2+)-ATPase, isoform 4 |
| Pp02 | [962761..964952](http://www.rosaceae.org/gb/gbrowse/prunus_persica?name=scaffold_2:962761..964952&enable=NCBI%20Sequence%20Alignments) | HD | ppa015742m | AT4G08580.1 | Microfibrillar-associated protein-related |
| Pp02 | [9866881..9871438](http://www.rosaceae.org/gb/gbrowse/prunus_persica?name=scaffold_2:9866881..9871438&enable=NCBI%20Sequence%20Alignments) | HD, Sorbitol | ppa021778m | ATMG00860.1 | DNA/RNA polymerases superfamily protein |
| Pp03 | [20635345..20638079](http://www.rosaceae.org/gb/gbrowse/prunus_persica?name=scaffold_3:20635345..20638079&enable=NCBI%20Sequence%20Alignments) | BD | ppa002530m | AT5G40380.1 | Cysteine-rich RLK (RECEPTOR-like protein kinase) 42 |
| Pp03 | [4002097..4006716](http://www.rosaceae.org/gb/gbrowse/prunus_persica?name=scaffold_3:4002097..4006716&enable=NCBI%20Sequence%20Alignments) | HD | ppa024192m | AT1G50830.1 | Aminotransferase-like, plant mobile domain family protein |
| Pp03 | [5657462..5661579](http://www.rosaceae.org/gb/gbrowse/prunus_persica?name=scaffold_3:5657462..5661579&enable=NCBI%20Sequence%20Alignments) | HD | ppa022649m | AT3G14470.1 | NB-ARC domain-containing disease resistance protein |
| Pp04 | [15567730..15574374](http://www.rosaceae.org/gb/gbrowse/prunus_persica?name=scaffold_4:15567730..15574374&enable=NCBI%20Sequence%20Alignments) | BD, HD | ppa021048m | AT1G11310.1 | Seven transmembrane MLO family protein |
| Pp04 | [15743100..15746688](http://www.rosaceae.org/gb/gbrowse/prunus_persica?name=scaffold_4:15743100..15746688&enable=NCBI%20Sequence%20Alignments) | BD, HD | ppa016424m | AT1G26540.1 | Agenet domain-containing protein |
| Pp04 | [18422741..18426896](http://www.rosaceae.org/gb/gbrowse/prunus_persica?name=scaffold_4:18422741..18426896&enable=NCBI%20Sequence%20Alignments) | BD, HD | ppa003549m | AT5G12860.1 | Dicarboxylate transporter 1 |
| Pp04 | [18495283..18497121](http://www.rosaceae.org/gb/gbrowse/prunus_persica?name=scaffold_4:18495283..18497121&enable=NCBI%20Sequence%20Alignments) | BD, HD | ppa007944m | AT1G49390.1 | 2-oxoglutarate (2OG) and Fe(II)-dependent superfamily protein |
| Pp04 | [18519782..18524365](http://www.rosaceae.org/gb/gbrowse/prunus_persica?name=scaffold_4:18519782..18524365&enable=NCBI%20Sequence%20Alignments) | BD, HD | ppa024381m | AT5G18350.1 | Disease resistance protein (TIR-NBS-LRR class) family |
| Pp04 | [18546574..18548682](http://www.rosaceae.org/gb/gbrowse/prunus_persica?name=scaffold_4:18546574..18548682&enable=NCBI%20Sequence%20Alignments) | HD, | ppa025636m | AT2G41900.1 | CCCH-type zinc finger protein with ARM repeat domain |
| Pp04 | [18584006..18587101](http://www.rosaceae.org/gb/gbrowse/prunus_persica?name=scaffold_4:18584006..18587101&enable=NCBI%20Sequence%20Alignments) | HD, Sorbitol, TS | ppa011093m | AT5G19855.1 | Chaperonin-like RbcX protein |
| Pp04 | [18589213..18593156](http://www.rosaceae.org/gb/gbrowse/prunus_persica?name=scaffold_4:18589213..18593156&enable=NCBI%20Sequence%20Alignments) | HD, Sorbitol, TS | ppa011397m | AT1G54250.1 | RNA polymerase Rpb8 |
| Pp04 | [18654418..18655887](http://www.rosaceae.org/gb/gbrowse/prunus_persica?name=scaffold_4:18654418..18655887&enable=NCBI%20Sequence%20Alignments) | HD, Sorbitol, TS | ppb017730m | AT1G51340.2 | MATE efflux family protein |
| Pp04 | [18740972..18743852](http://www.rosaceae.org/gb/gbrowse/prunus_persica?name=scaffold_4:18740972..18743852&enable=NCBI%20Sequence%20Alignments) | HD, Sorbitol, TS | ppa021927m | AT1G51405.1 | Myosin-related |
| Pp04 | [18769655..18771374](http://www.rosaceae.org/gb/gbrowse/prunus_persica?name=scaffold_4:18769655..18771374&enable=NCBI%20Sequence%20Alignments) | HD, Sorbitol, TS | ppa023872m | AT1G75280.1 | NmrA-like negative transcriptional regulator family protein |
| Pp04 | [18861347..18863258](http://www.rosaceae.org/gb/gbrowse/prunus_persica?name=scaffold_4:18861347..18863258&enable=NCBI%20Sequence%20Alignments) | HD, Sorbitol, TS | ppa022560m | AT2G04865.1 | Aminotransferase-like, plant mobile domain family protein |
| Pp04 | [19396581..19399569](http://www.rosaceae.org/gb/gbrowse/prunus_persica?name=scaffold_4:19396581..19399569&enable=NCBI%20Sequence%20Alignments) | HD, Sorbitol, TS | ppa020850m | AT1G64260.1 | MuDR family transposase |
| Pp04 | [19905035..19907727](http://www.rosaceae.org/gb/gbrowse/prunus_persica?name=scaffold_4:19905035..19907727&enable=NCBI%20Sequence%20Alignments) | HD, Sorbitol, TS | ppa027136m | AT5G65560.1 | Pentatricopeptide repeat (PPR) superfamily protein |
| Pp04 | [20164531..20165799](http://www.rosaceae.org/gb/gbrowse/prunus_persica?name=scaffold_4:20164531..20165799&enable=NCBI%20Sequence%20Alignments) | HD, Sorbitol | ppa023762m | AT4G20860.1 | FAD-binding Berberine family protein |
| Pp04 | [5725127..5727408](http://www.rosaceae.org/gb/gbrowse/prunus_persica?name=scaffold_4:5725127..5727408&enable=NCBI%20Sequence%20Alignments) | Anthoc | ppb015985m | AT2G23980.1 | Cyclic nucleotide-gated channel 6 |
| Pp04 | [8996062..8997148](http://www.rosaceae.org/gb/gbrowse/prunus_persica?name=scaffold_4:8996062..8997148&enable=NCBI%20Sequence%20Alignments) | HD | ppa025081m | AT1G01980.1 | FAD-binding Berberine family protein |
| Pp04 | [9039533..9044737](http://www.rosaceae.org/gb/gbrowse/prunus_persica?name=scaffold_4:9039533..9044737&enable=NCBI%20Sequence%20Alignments) | HD | ppa022208m | AT3G14840.2 | Leucine-rich repeat transmembrane protein kinase |
| Pp05 | [14994506..14997190](http://www.rosaceae.org/gb/gbrowse/prunus_persica?name=scaffold_5:14994506..14997190&enable=NCBI%20Sequence%20Alignments) | HD | ppa007344m | AT5G40240.2 | Nodulin MtN21 /EamA-like transporter family protein |
| Pp05 | [276075..278590](http://www.rosaceae.org/gb/gbrowse/prunus_persica?name=scaffold_5:276075..278590&enable=NCBI%20Sequence%20Alignments) | HD | ppa018968m | AT1G76620.1 | Protein of unknown function, DUF547 |
| Pp05 | [463584..470109](http://www.rosaceae.org/gb/gbrowse/prunus_persica?name=scaffold_5:463584..470109&enable=NCBI%20Sequence%20Alignments) | HD | ppa002571m | AT1G76550.1 | Phosphofructokinase family protein |
| Pp05 | [479669..482411](http://www.rosaceae.org/gb/gbrowse/prunus_persica?name=scaffold_5:479669..482411&enable=NCBI%20Sequence%20Alignments) | HD | ppa026999m | AT4G38180.1 | FAR1-related sequence 5 |
| Pp05 | [4918389..4925225](http://www.rosaceae.org/gb/gbrowse/prunus_persica?name=scaffold_5:4918389..4925225&enable=NCBI%20Sequence%20Alignments) | HD | ppa003016m | AT4G18030.1 | S-adenosyl-L-methionine-dependent superfamily protein |
| Pp05 | [4971560..4973074](http://www.rosaceae.org/gb/gbrowse/prunus_persica?name=scaffold_5:4971560..4973074&enable=NCBI%20Sequence%20Alignments) | HD | ppa018546m | AT1G50920.1 | Nucleolar GTP-binding protein |
| Pp05 | [5173913..5176862](http://www.rosaceae.org/gb/gbrowse/prunus_persica?name=scaffold_5:5173913..5176862&enable=NCBI%20Sequence%20Alignments) | HD | ppa001175m | AT1G17070.1 | GC-rich sequence DNA-binding factor-like protein domain |
| Pp05 | [519684..522366](http://www.rosaceae.org/gb/gbrowse/prunus_persica?name=scaffold_5:519684..522366&enable=NCBI%20Sequence%20Alignments) | HD | ppa017924m | AT1G20925.1 | Auxin efflux carrier family protein |
| Pp05 | [5198367..5202583](http://www.rosaceae.org/gb/gbrowse/prunus_persica?name=scaffold_5:5198367..5202583&enable=NCBI%20Sequence%20Alignments) | HD | ppa000588m | AT1G05490.1 | Chromatin remodeling 31 |
| Pp06 | [10460008..10460634](http://www.rosaceae.org/gb/gbrowse/prunus_persica?name=scaffold_6:10460008..10460634&enable=NCBI%20Sequence%20Alignments) | HD, Flavonoids | ppa019114m | AT3G62020.1 | Germin-like protein 10 |
| Pp06 | [10602903..10605060](http://www.rosaceae.org/gb/gbrowse/prunus_persica?name=scaffold_6:10602903..10605060&enable=NCBI%20Sequence%20Alignments) | HD, Flavonoids, TS | ppa022395m | ATMG00860.1 | DNA/RNA polymerases superfamily protein |
| Pp06 | [10606327..10607559](http://www.rosaceae.org/gb/gbrowse/prunus_persica?name=scaffold_6:10606327..10607559&enable=NCBI%20Sequence%20Alignments) | HD, Flavonoids, TS | ppa010337m | AT5G22380.1 | NAC domain containing protein 90 |
| Pp06 | [10733407..10734894](http://www.rosaceae.org/gb/gbrowse/prunus_persica?name=scaffold_6:10733407..10734894&enable=NCBI%20Sequence%20Alignments) | HD, Flavonoids | ppa021329m | AT3G10230.1 | Lycopene cyclase |
| Pp06 | [10822570..10828704](http://www.rosaceae.org/gb/gbrowse/prunus_persica?name=scaffold_6:10822570..10828704&enable=NCBI%20Sequence%20Alignments) | HD, Flavonoids | ppa018907m | AT4G29090.1 | Ribonuclease H-like superfamily protein |
| Pp06 | [11001690..11004541](http://www.rosaceae.org/gb/gbrowse/prunus_persica?name=scaffold_6:11001690..11004541&enable=NCBI%20Sequence%20Alignments) | HD, Flavonoids | ppa020368m | AT5G42260.1 | Beta glucosidase 12 |
| Pp06 | [11016434..11017621](http://www.rosaceae.org/gb/gbrowse/prunus_persica?name=scaffold_6:11016434..11017621&enable=NCBI%20Sequence%20Alignments) | HD, Flavonoids | ppa026662m | AT3G52500.1 | Eukaryotic aspartyl protease family protein |
| Pp06 | [28043074..28046128](http://www.rosaceae.org/gb/gbrowse/prunus_persica?name=scaffold_6:28043074..28046128&enable=NCBI%20Sequence%20Alignments) | HD, Sorbitol | ppa006828m | AT3G04460.1 | Peroxin-12 |
| Pp06 | [4756263..4760831](http://www.rosaceae.org/gb/gbrowse/prunus_persica?name=scaffold_6:4756263..4760831&enable=NCBI%20Sequence%20Alignments) | HD | ppa026856m | ATMG00860.1 | DNA/RNA polymerases superfamily protein |
| Pp06 | [7901285..7903495](http://www.rosaceae.org/gb/gbrowse/prunus_persica?name=scaffold_6:7901285..7903495&enable=NCBI%20Sequence%20Alignments) | HD, Flavonoids | ppa007943m | AT2G36870.1 | Xyloglucan endotransglucosylase/hydrolase 32 |
| Pp06 | [7911196..7914781](http://www.rosaceae.org/gb/gbrowse/prunus_persica?name=scaffold_6:7911196..7914781&enable=NCBI%20Sequence%20Alignments) | HD, Flavonoids | ppa024389m | AT5G36930.2 | Disease resistance protein (TIR-NBS-LRR class) family |
| Pp06 | [8000326..8001998](http://www.rosaceae.org/gb/gbrowse/prunus_persica?name=scaffold_6:8000326..8001998&enable=NCBI%20Sequence%20Alignments) | HD, Flavonoids | ppa024782m | AT2G28120.1 | Major facilitator superfamily protein |
| Pp06 | [8225577..8230643](http://www.rosaceae.org/gb/gbrowse/prunus_persica?name=scaffold_6:8225577..8230643&enable=NCBI%20Sequence%20Alignments) | HD, Flavonoids | ppa001257m | AT2G27950.1 | Ring/U-Box superfamily protein |
| Pp06 | [8232072..8232989](http://www.rosaceae.org/gb/gbrowse/prunus_persica?name=scaffold_6:8232072..8232989&enable=NCBI%20Sequence%20Alignments) | HD, Flavonoids | ppa024155m | AT1G08170.1 | Histone superfamily protein |
| Pp06 | [8333981..8338484](http://www.rosaceae.org/gb/gbrowse/prunus_persica?name=scaffold_6:8333981..8338484&enable=NCBI%20Sequence%20Alignments) | HD, Flavonoids | ppa021011m | AT5G22860.1 | Serine carboxypeptidase S28 family protein |
| Pp06 | [8367747..8371683](http://www.rosaceae.org/gb/gbrowse/prunus_persica?name=scaffold_6:8367747..8371683&enable=NCBI%20Sequence%20Alignments) | HD, Flavonoids | ppa004651m | AT5G22850.1 | Eukaryotic aspartyl protease family protein |
| Pp07 | [14885315..14895639](http://www.rosaceae.org/gb/gbrowse/prunus_persica?name=scaffold_7:14885315..14895639&enable=NCBI%20Sequence%20Alignments) | HD | ppa000451m | AT3G53090.2 | Ubiquitin-protein ligase 7 |
| Pp07 | [14944296..14948721](http://www.rosaceae.org/gb/gbrowse/prunus_persica?name=scaffold_7:14944296..14948721&enable=NCBI%20Sequence%20Alignments) | HD | ppa004123m | AT3G53110.1 | P-loop containing nucleoside triphosphate superfamily protein |
| Pp07 | [16142834..16145927](http://www.rosaceae.org/gb/gbrowse/prunus_persica?name=scaffold_7:16142834..16145927&enable=NCBI%20Sequence%20Alignments) | HD | ppa024687m | AT2G23530.1 | Zinc-finger domain of monoamine-oxidase A repressor R1 |
| Pp07 | [18388553..18390591](http://www.rosaceae.org/gb/gbrowse/prunus_persica?name=scaffold_7:18388553..18390591&enable=NCBI%20Sequence%20Alignments) | HD | ppa006321m | AT5G65960.1 | GTP binding |
| Pp07 | [18508817..18511419](http://www.rosaceae.org/gb/gbrowse/prunus_persica?name=scaffold_7:18508817..18511419&enable=NCBI%20Sequence%20Alignments) | HD, RI | ppa008685m | AT3G51520.1 | Diacylglycerol acyltransferase family |
| Pp07 | [18648848..18650712](http://www.rosaceae.org/gb/gbrowse/prunus_persica?name=scaffold_7:18648848..18650712&enable=NCBI%20Sequence%20Alignments) | HD, RI | ppa003852m | AT2G26440.1 | Plant invertase/pectin methylesterase inhibitor superfamily |
| Pp07 | [18676141..18684484](http://www.rosaceae.org/gb/gbrowse/prunus_persica?name=scaffold_7:18676141..18684484&enable=NCBI%20Sequence%20Alignments) | HD | ppa000098m | AT3G14270.1 | Phosphatidylinositol-4-phosphate 5-kinase family protein |
| Pp07 | [18751831..18757844](http://www.rosaceae.org/gb/gbrowse/prunus_persica?name=scaffold_7:18751831..18757844&enable=NCBI%20Sequence%20Alignments) | HD, RI | ppa001535m | AT3G43190.1 | Sucrose synthase 4 |
| Pp07 | [18753725..18757844](http://www.rosaceae.org/gb/gbrowse/prunus_persica?name=scaffold_7:18753725..18757844&enable=NCBI%20Sequence%20Alignments) | HD, RI | ppa001537m | AT3G43190.1 | Sucrose synthase 4 |
| Pp07 | [18840993..18844653](http://www.rosaceae.org/gb/gbrowse/prunus_persica?name=scaffold_7:18840993..18844653&enable=NCBI%20Sequence%20Alignments) | HD, RI | ppa003282m | AT2G40700.1 | P-loop containing nucleoside triphosphate superfamily protein |
| Pp07 | [19333781..19336099](http://www.rosaceae.org/gb/gbrowse/prunus_persica?name=scaffold_7:19333781..19336099&enable=NCBI%20Sequence%20Alignments) | HD, RI | ppa013187m | AT4G26060.1 | Ribosomal protein L18ae family |
| Pp07 | [19405800..19409668](http://www.rosaceae.org/gb/gbrowse/prunus_persica?name=scaffold_7:19405800..19409668&enable=NCBI%20Sequence%20Alignments) | HD, RI | ppa007796m | AT5G67580.2 | Homeodomain-like/winged-helix DNA-binding family protein |
| Pp07 | [19508173..19511574](http://www.rosaceae.org/gb/gbrowse/prunus_persica?name=scaffold_7:19508173..19511574&enable=NCBI%20Sequence%20Alignments) | HD, RI | ppa021529m | AT3G50150.1 | Plant protein of unknown function (DUF247) |
| Pp07 | [19521492..19523870](http://www.rosaceae.org/gb/gbrowse/prunus_persica?name=scaffold_7:19521492..19523870&enable=NCBI%20Sequence%20Alignments) | HD, RI | ppa009258m | AT3G47560.1 | Alpha/beta-Hydrolases superfamily protein |
| Pp07 | [19521492..19523870](http://www.rosaceae.org/gb/gbrowse/prunus_persica?name=scaffold_7:19521492..19523870&enable=NCBI%20Sequence%20Alignments) | HD, RI | ppa009975m | AT3G47560.1 | Alpha/beta-Hydrolases superfamily protein |
| Pp07 | [19540330..19542736](http://www.rosaceae.org/gb/gbrowse/prunus_persica?name=scaffold_7:19540330..19542736&enable=NCBI%20Sequence%20Alignments) | HD, RI | ppa021859m | AT4G29740.2 | Cytokinin oxidase 4 |
| Pp07 | [22672600..22674933](http://www.rosaceae.org/gb/gbrowse/prunus_persica?name=scaffold_7:22672600..22674933&enable=NCBI%20Sequence%20Alignments) | HD | ppa019788m | AT5G08310.1 | Tetratricopeptide repeat (TPR)-like superfamily protein |
| Pp07 | [7470206..7471971](http://www.rosaceae.org/gb/gbrowse/prunus_persica?name=scaffold_7:7470206..7471971&enable=NCBI%20Sequence%20Alignments) | HD | ppa019552m | AT3G56970.1 | Basic helix-loop-helix (bHLH) DNA-binding superfamily protein |
| Pp07 | [7829460..7837572](http://www.rosaceae.org/gb/gbrowse/prunus_persica?name=scaffold_7:7829460..7837572&enable=NCBI%20Sequence%20Alignments) | HD | ppa000276m | AT3G57060.1 | Binding |
| Pp08 | [1267101..1271827](http://www.rosaceae.org/gb/gbrowse/prunus_persica?name=scaffold_8:1267101..1271827&enable=NCBI%20Sequence%20Alignments) | HD | ppa015000m | ATMG00860.1 | DNA/RNA polymerases superfamily protein |
| Pp08 | [15785774..15787316](http://www.rosaceae.org/gb/gbrowse/prunus_persica?name=scaffold_8:15785774..15787316&enable=NCBI%20Sequence%20Alignments) | HD | ppb022611m | AT4G22780.1 | ACT domain repeat 7 |
| Pp08 | [17929397..17931430](http://www.rosaceae.org/gb/gbrowse/prunus_persica?name=scaffold_8:17929397..17931430&enable=NCBI%20Sequence%20Alignments) | HD, TS | ppa009839m | AT3G07370.1 | Carboxyl terminus of HSC70-interacting protein |
| Pp08 | [18084960..18086676](http://www.rosaceae.org/gb/gbrowse/prunus_persica?name=scaffold_8:18084960..18086676&enable=NCBI%20Sequence%20Alignments) | HD, TS | ppa004095m | AT3G25180.1 | Cytochrome P450, family 82, subfamily G, polypeptide 1 |
| Pp08 | [18116841..18118176](http://www.rosaceae.org/gb/gbrowse/prunus_persica?name=scaffold_8:18116841..18118176&enable=NCBI%20Sequence%20Alignments) | HD, Sorbitol, TS | ppa022619m | AT3G29575.4 | ABI five binding protein 3 |
| Pp08 | [18218585..18222930](http://www.rosaceae.org/gb/gbrowse/prunus_persica?name=scaffold_8:18218585..18222930&enable=NCBI%20Sequence%20Alignments) | HD, Sorbitol, TS | ppa003075m | AT3G07180.1 | GPI transamidase component PIG-S-related |
| Pp08 | [18243363..18245829](http://www.rosaceae.org/gb/gbrowse/prunus_persica?name=scaffold_8:18243363..18245829&enable=NCBI%20Sequence%20Alignments) | HD, Sorbitol, TS | ppa024636m | AT3G07140.1 | GPI transamidase component Gpi16 subunit family protein |
| Pp08 | [18308467..18316718](http://www.rosaceae.org/gb/gbrowse/prunus_persica?name=scaffold_8:18308467..18316718&enable=NCBI%20Sequence%20Alignments) | HD, Sorbitol, TS | ppa021659m | AT3G43920.2 | Dicer-like 3 |
| Pp08 | [2985727..2988409](http://www.rosaceae.org/gb/gbrowse/prunus_persica?name=scaffold_8:2985727..2988409&enable=NCBI%20Sequence%20Alignments) | HD | ppa024810m | AT2G47430.1 | Signal transduction histidine kinase |

Abbreviations: HD, harvest date; TS, total sugars; BD, blooming date; anthoc, anthocyanins; RAC, relative antioxidant capacity; RI, ripening index
